# Transcriptome-Wide Identification of an Aurone Glycosyltransferase with Glycosidase Activity from *Ornithogalum saundersiae*

**DOI:** 10.3390/genes9070327

**Published:** 2018-06-28

**Authors:** Shuai Yuan, Ming Liu, Yan Yang, Jiu-Ming He, Ya-Nan Wang, Jian-Qiang Kong

**Affiliations:** Institute of Materia Medica, Chinese Academy of Medical Sciences & Peking Union Medical College (State Key Laboratory of Bioactive Substance and Function of Natural Medicines & Ministry of Health Key Laboratory of Biosynthesis of Natural Products), Beijing 100050, China; yuanshuai@imm.ac.cn (S.Y.); lm406977545@outlook.com (M.L.); yangyan@imm.ac.cn (Y.Y.); hejiuming@imm.ac.cn (J.-M.H.); wangyanan@imm.ac.cn (Y.-N.W.)

**Keywords:** aurones, sulfuretin, glycosyltransferase, *Ornithogalum saundersiae*

## Abstract

Aurone glycosides display a variety of biological activities. However, reports about glycosyltransferases (GTs) responsible for aurones glycosylation are limited. Here, the transcriptome-wide discovery and identification of an aurone glycosyltransferase with glycosidase activity is reported. Specifically, a complementary DNA (cDNA), designated as OsUGT1, was isolated from the plant *Ornithogalum saundersiae* based on transcriptome mining. Conserved domain (CD)-search speculated OsUGT1 as a flavonoid GT. Phylogenetically, OsUGT1 is clustered as the same phylogenetic group with a putative 5,6-dihydroxyindoline-2-carboxylic acid (*cyclo*-DOPA) 5-*O*-glucosyltransferase, suggesting OsUGT1 may be an aurone glycosyltransferase. The purified OsUGT1 was therefore used as a biocatalyst to incubate with the representative aurone sulfuretin. In vitro enzymatic analyses showed that OsUGT1 was able to catalyze sulfuretin to form corresponding monoglycosides, suggesting OsUGT1 was indeed an aurone glycosyltransferase. OsUGT1 was observed to be a flavonoid GT, specific for flavonoid substrates. Moreover, OsUGT1 was demonstrated to display transglucosylation activity, transferring glucosyl group to sulfuretin via *o*-Nitrophenyl-β-d-glucopyranoside (*o*NP-β-Glc)-dependent fashion. In addition, OsUGT1-catalyzed hydrolysis was observed. This multifunctionality of OcUGT1 will broaden the application of OcUGT1 in glycosylation of aurones and other flavonoids.

## 1. Introduction

**Figure 1:**
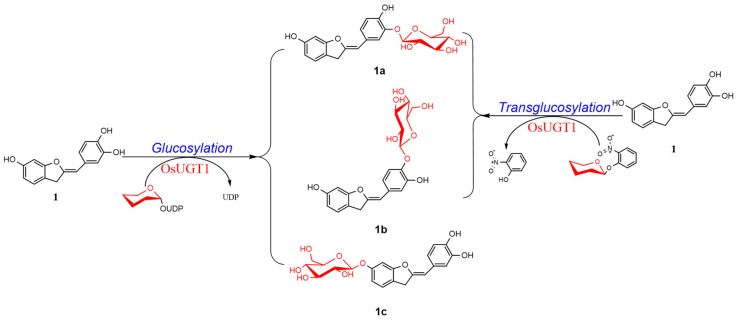
OsUGT1-catalyzed biosynthesis of sulfuretin glucosides (**1a**, **1b** and **1c**) via glucosylation or transglucosylation reaction. UDP: uridine diphosphate; OsUGT1: glycosyltransferase from *Ornithogalum saundersiae*.

Aurones, represented by sulfuretin (Figure 1, 1) and sulfurein (Figure 1, 1c), are minor tricyclic flavonoids that consist of a benzofuranone ring linked through a carbon-carbon double bond to a phenyl moiety (Figure 1) [1]. Besides lending flowers a bright yellow pigment, aurones function as phytoalexins against infections [2]. Aurones distribute widely among the plant kingdom and two aurone types can be distinguished, 4-hydroxyaurones and 4-deoxyaurones [1]. Aurones show a remarkable spectrum of biological activities, like antioxidant, antitumor, antimicrobial, antiviral and anti-inflammatory activities, making them interesting scaffolds for the design of potential therapeutic agents [1,3]. Thus, many efforts have been made for synthetic modifications of aurone scaffolds, such as glycosylation, hydroxylation and methoxylation [1].

Among these modifications, glycosylation of aurones have attracted much interest because these resulting glycosylated aurones display enhanced and advantageous properties, like neuroprotective [4], antioxidant [5,6,7,8,9], anti-inflammatory [10] and leishmanicidal activities [11]. Theoretically, glycosylations of aurones may be achieved by chemical or enzymatical syntheses. Chemical approaches generally require multiple protection and de-protection steps in order to achieve regio- and stereo-selectivity. Moreover, chemical glycosylations involve the use of toxic and hazardous chemicals, which may pose potential environmental and biological risks. For such reasons, glycosyltransferases (GTs)-catalyzed glycosylations become increasingly important enzymatic synthesis due to its high regio-selectivity without the need of any protecting groups. The enzymatic glucosylation of 4-deoxyaurones was firstly achieved by Halbwirth et al., 1997 [12]. Sulfuretin 6-glucoside, namely sulfurein (1c), was yielded after incubation of sulfuretin (1) with enzyme preparation from *Coreopsis grandiflora*. However, the biocatalyst used in this glucosylation reaction was enzyme extract of *C. grandiflora* but not a purified glucosyltransferase [12]. Sakakibara et al. provided a gene encoding an aurone glycosyltransferase with an activity of transferring a glycosyl group to an aurone [13]. However, this aurone GT required expensive uridine diphosphate (UDP)-sugar as the sugar donor for glycosylations of aurones, which limited its extensive applications [13]. Herein, the transcriptome-wide discovery and identification of an aurone glycosyltransferase OsUGT1 with glycosidase activity was described, which meant OsUGT1 was able to glycosylate aurones using either expensive UDP-sugars or the cheap alternatives as sugar donors. Specifically, a complementary DNA (cDNA), designated as OsUGT1, coding for an aurone GT was firstly isolated from *Ornithogalum saundersiae* based on transcriptome mining. The recombinant OsUGT1 was then determined to have the ability to glucosylate sulfuretin (1) to form three monoglucosides using UDP-d-glucose (UDP-Glc) as a sugar donor. In addition, OsUGT1 displayed catalytic promiscuity, transferring sugar group to diverse flavonoids from UDP-Glc. Besides being a glycosyltransferase, OsUGT1 was demonstrated to display transglycosylase activity, forming sulfuretin glucosides using cheap aryl glycosides as sugar donor by transglycosylation. Moreover, OsUGT1-catalyzed hydrolysis activity was observable. This multifunctionality thus broadened the potential applications of OsUGT1 in glycosylation of aurones and other flavonoids. 

## 2. Materials and Methods

### 2.1. Chemicals

**Figure 2:**
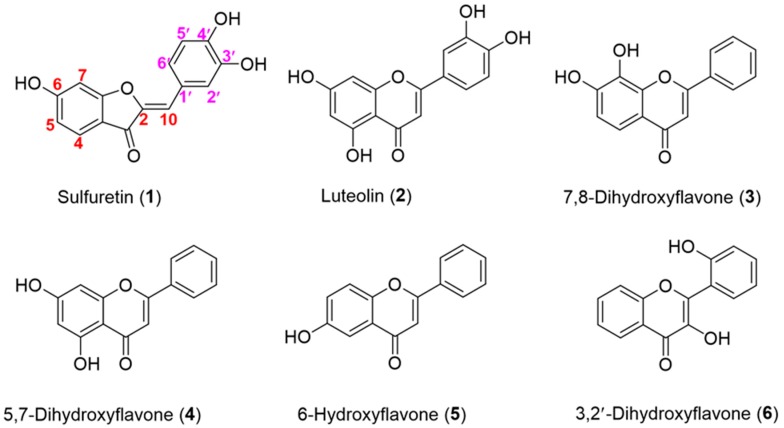
The detailed structures of 6 reactive substrates of OsUGT1.

A total of 41 compounds, including flavonoids (**1**–**7**), anthraquinones (**8**), steroids (**9**–**25**), terpenoids (**26**–**28**), phenolic acids (**29**–**33**) and alkaloids (**34**–**41**), were used as the sugar acceptors for OsUGT1-catalyzed glycosylation reactions (Figure 2, Table S1). Four UDP-activated nucleotides, namely UDP-Glc, UDP-*N*-acetylglucosamine (UDP-GlcNAc), UDP-D-xylose (UDP-Xyl) and UDP-l-arabinose (UDP-Ara), were used as the sugar donors. UDP-Glc and UDP-GlcNAc were obtained from Sigma-Aldrich Co. LLC (St. Louis, MO, USA). UDP-Xyl and UDP-Ara were enzymatically synthesized as described previously [14,15]. Two aryl-substituted glycosides, *o*-Nitrophenyl-β-d-glucopyranoside (*o*NP-β-Glc) and *o*-Nitrophenyl-β-d-galactopyranoside (*o*NP-β-Gal), were purchased from J&K Chemical Ltd (Shanghai, China) and applied as the substrates for transglucosylation or hydrolysis reaction [16]. The other chemicals were either reagents or analytic grade when available.

### 2.2. Retrieval of Unigenes encoding GT from Transcriptome Database of O. saundersiae

The transcriptome of *O. saundersiae* was sequenced previously and the resultant database was kept in our laboratory [17]. The unigenes in this database were first aligned by Blast X algorithm to protein databases, such as Non-Redundant Dataset (NR), Swiss-Prot, Kyoto Encyclopedia of Genes and Genomes (KEGG) and Cluster of Orthologous Groups (COG) (*e*-value < 0.00001) for functional annotation. The unigene with a complete open reading frame (ORF), and displaying high similarity to uridine diphosphate glycosyltransferases (UGTs), was selected as the candidate for further investigation. The process of unigenes retrieval was detailed as described previously [18,19].

### 2.3. cDNA Isolation of the Gene Encoding OsUGT1 from O. saundersiae

Fresh bulbs of *O. saundersiae* were collected to frozen in liquid N_2_ for RNA isolation. The purified RNA was then reverse transcribed into full-length cDNA. All procedures were carried out according to the manufacturer’s directions (ReverTra Plus kit, Toyobo Life Science, Shanghai, China).

The obtained reverse transcripts were used as the template for nested PCR assay with the candidate unigene-specific primers (Table S2). The resultant PCR product was separated by agarose gel electrophoresis and then inserted into the T-A cloning vector *pEASY*^TM^-Blunt (TransGen Co. Ltd, Beijing, China) (Table S3). The ligation mixture was transformed into strain *Trans*1-T1 (TransGen Co. Ltd, Beijing, China) for plasmid recombination. The resultant plasmid pEASY-OsUGT1 carrying the cDNA insert was subjected to sequencing (Table S3).

### 2.4. Bioinformatics Analyses of OsUGT1

To direct the heterologous expression and functional identification, bioinformatics analyses of OsUGT1, like the computation of various physical and chemical parameters, multiple sequence alignment and phylogenetic analysis, were performed as described previously [18]. In brief, the computation of the molecular weight and theoretical isoelectric point (pI) was performed in ExPASy ProtParam tool (http://web.expasy.org/protparam/). The conserved domain (CD)-search was run at the NCBI server (https://www.ncbi.nlm.nih.gov/) [20]. A phylogenetic tree was constructed using the neighbor-joining method with the MEGA7.0 program [21]. The reliability of the tree was measured by bootstrap analysis with 1000 replicates.

### 2.5. Heterologous Expression and Purification of OsUGT1

OsUGT1 gene amplified from pEASY-OsUGT1 was ligated into *Eco*R I/*Hin*d III-digested pET-28a (+) (Novagen, Madison, WI, USA) using seamless assembly cloning kit (CloneSmarter Technologies Inc., Houston, TX, USA) following the supplier recommendation. The resultant construct pET28a-OsUGT1 was transformed into the bacterial strain *Trans*etta (DE3) (TransGen Co. Ltd., Beijing, China). Cells containing pET28a-OsUGT1 were cultured in Luria Broth in the presence of chloramphenicol (34 μg/mL) and kanamycin (50 μg/mL) at 37 °C until the optical density at 600 nm reached 0.6 (OD_600_ = 0.6). At this point, the production of the recombinant OsUGT1 was induced by the addition of isopropyl-d-thiogalactopyranoside (IPTG) to a final concentration of 0.2 mM. Fifteen hours at 18 °C after induction, cell pellets were harvested by centrifugation (10,000× *g*, 1 min) and then disrupted using a sonicator. The cell debris was separated by centrifugation at 10,000× *g* for 2 min at 4 °C. The resulting cell-free supernatant was analyzed via sodium dodecyl sulfate polyacrylamide gel electrophoresis (SDS-PAGE) followed by western-blotting analysis with an anti-His-tag antibody.

The bacterially produced OsUGT1 protein was batch purified by immobilized metal ion affinity chromatography according to the manufacturer’s recommendations (CWBio, Beijing, China). The concentration of purified OsUGT1 was determined using Bio-Rad protein assay with bovine serum albumin as a standard.

### 2.6. OsUGT1-Catalyzed Glycosylation Assays

The cell-free supernatant containing the recombinant OsUGT1 or the purified OsUGT1 was used as a biocatalyst for glycosylation reaction, which was performed at 37 °C for 60 min in 100 μL of 20 mM of phosphate buffer solution (PBS buffer, pH 8.0) supplemented with 1 mM sugar donor, 1 mM sugar acceptor and the biocatalyst. The formation of new glycosides was monitored by high performance liquid chromatography (HPLC). The HPLC conditions have been detailed as described by Yuan et al. [16] and Liu and Kong [17]. The resulting glycosides were collected by reversed-phase HPLC and then injected into a TripleTOF 5600 MS instrument (AB Sciex, Concord, ON, Canada) operated in positive mode with a DuoSpray ion source. The exact structures of aurone glycosides were well characterized by nuclear magnetic resonance(NMR) analysis, which was performed as described by Yuan et al. [16].

### 2.7. Enzymatic Characterization

The effects of pH, temperature and metal ions on the glycosylation activity of OsUGT1 towards 7,8-dihydroxyflavone (3) were conducted using the purified OsUGT1 as the biocatalyst. The effect of pH on the activities of OsUGT1 was determined by monitoring OsUGT1 activities in 10 mM of buffers with varied pH values including acetate buffer (pH 4–6), PBS buffer (pH 6–9) and carbonate buffer solution (CBS buffer, pH 9–11). The influence of temperature on OsUGT1-catalyzed glucosylation was tested in optimum pH at selected temperatures from 0 to 70 °C. The role of metal ions in the activity of OsUGT1 was assessed under the optimum pH and temperature. The additives used in this study were the salts of Mg^2+^, Li^+^, Ca^2+^, Mn^2+^, Zn^2+^, Co^2+^, Cu^2+^ and ethylenediaminetetraacetic acid(EDTA)-2Na at 5 mM final concentration.

The kinetic parameters, namely Michaelis constant (*K*_m_) and maximum velocity (*V*_max_) values, of OsUGT1-catalyzed glucosylation for sulfuretin (1) and 7,8-dihydroxyflavone (3) were determined by varying the concentrations of sulfuretin (1) or 7,8-dihydroxyflavone (3) from 0 to 1 mM, at a fixed UDP-Glc concentration of 1 mM. Data were transformed and plotted as Lineweaver-Burk graphs for the calculation of *K_m_* and *V*_max_ values.

### 2.8. OsUGT1-Mediated Transglucosylation Assay

The transglucosylation activity of OsUGT1 towards sulfuretin (1) was determined as previously described [16]. An aryl-substituted glycoside *o*NP-β-Glc was used as a sugar donor. The reaction was performed at 37 °C for 2 h and monitored by HPLC.

### 2.9. OsUGT1-Assisted Hydrolysis Assay

OsUGT1-assisted hydrolysis assay was conducted at 37 °C for 2 h, as introduced by Yuan et al. [16]. A glycoside *o*NP-β-Gal was chosen as the hydrolytic substrate. The hydrolytic activity was monitored by measuring *o*-nitrophenol (*o*NP) release using HPLC.

## 3. Results

### 3.1. cDNA Isolation of OsUGT1 from O. saundersiae

*Ornithogalum saundersiae* is a medicinal plant rich in glycosides, suggesting this species contains glycosyltransferase responsible for the biosynthesis of glycosides [22,23]. Hence, the transcriptome sequencing of *O. saundersiae* was performed previously in our laboratory [17]. The extensive transcriptome mining was carried out with the aim of retrieving unigenes encoding GTs in this investigation. Unigene 28733, 1714 bp in length, was thus retrieved from the transcriptome database and its encoding protein was predicted to share high similarity with UGT. Moreover, unigene 28733 has a complete open reading frame of 1431 bases. The ATG start codon is preceded by a 5′-untranslated region (5′-UTR) of 66 nucleotides, while the annotated stop codon TGA locates in position 1495, immediately followed by a 3′-UTR of 217 bp. Therefore, unigene 28733 was selected as the candidate unigene for further functional characterization.

Firstly, a full-length cDNA corresponding to unigene 28733 was isolated from *O. saundersiae* by nested PCR assays. An intense band with ca. 1.5 kb was generated, as visualized on an agarose gel (Figure S1). Subsequently, the amplicon was inserted into *pEASY*-Blunt plasmid to generate a recombinant vector pEASY-OsUGT1 for sequencing (Table S3). Results indicated that the PCR fragment was exactly identical to that of unigene 28733, confirming unigene 28733 was a *bona fide* gene in *O. saundersiae* genome. The obtained cDNA was thus designated as OsUGT1 for convenience hereinafter and submitted to GenBank as accession number MH191373.

### 3.2. OsUGT1 was Predicted to Encode a Flavonoid Glycosyltransferase

The 1431-bp OsUGT1 encodes a peptide of 476 residues with a predicted molecular mass of 53.4 kDa and a pI of 5.95. OsUGT1 was deduced to display highest homology (63% identity, E = 0) to UGT 92A1-like protein of *Asparagus officinalis* (accession No XP_020249519.1) by running BlastP search.

The identification of conserved domain is deemed as an efficient tool to predict the function of a putative protein [20]. Hence, CD-search of OsUGT1 using BlastP heuristics was performed with the aim to identify the putative function of OsUGT1. At least three CD hits, like UDP-glucoronosyl/UDP-glucosyltransferase family protein (PLN02863) (Figure S2A), UDP:flavonoid glycosyltransferase YjiC, YdhE family (COG1819) (Figure S2B) and MGT family glycosyltransferase (TIGR01426) (Figure S2C), were obtained using OsUGT1 as the query sequence. PLN02863 CD was 477 bp long and contained a characteristic signature sequence, namely a plant secondary product glycosyltransferase (PSPG) motif, close to the C-terminal end (Figure S2A). The complete PSPG motif consists of 44 amino acid residues and is regarded as the nucleotide-diphosphate-sugar binding site [24]. The presence of PSPG motif revealed the possible role of OsUGT1 in secondary metabolism [24]. The COG1819 CD was 406 aa in length and the proteins containing COG1819 motif was believed to involve in the glycosylation of flavonoids (Figure S2B). The third CD is macroside glycosyltransferase (MGT) domain with accession number of TIGR01426 (Figure S2C). Many GTs harboring MGT domain are from microbe [25]. They are observed to be responsible for the glucosylation of macrolide antibiotics [25]. Moreover, these MGT domain-containing GTs were reported to be able to glucosylate a variety of flavonoids [25]. Together, we can basically speculate that OsUGT1 is a flavonoids glycosyltransferase based on the CD prediction results.

### 3.3. OsUGT1 was Clustered Phylogenetically to a New Group of Flavonoid Glycosyltransferases

**Figure 3:**
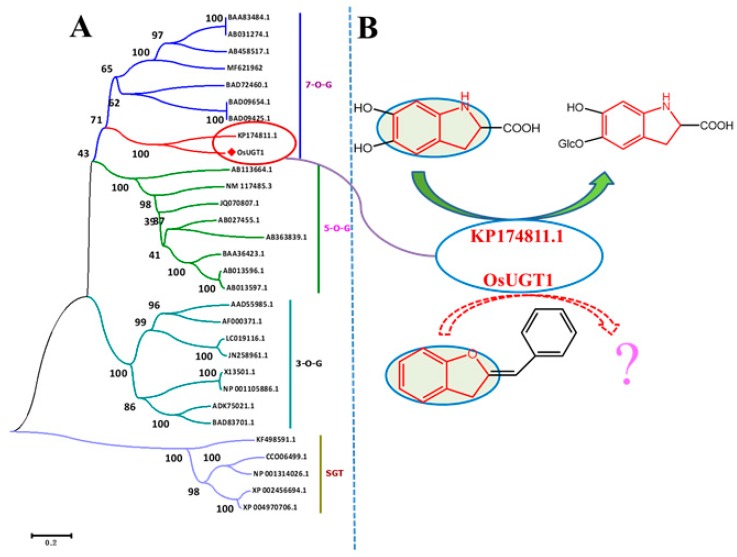
(**A**) Phylogenetic analysis of OsUGT1 with other GTs of known function. The deduced amino acid sequences were aligned with CLUSTAL X Version 2.0. The tree was constructed by the neighbor-joining method of MEGA 7.0. Numbers indicate the bootstrap values of 1000 replicates. The scale bar represents 0.2 amino acid substitutions per site. Different glycosyltransferases are clustered into 4 clads with varied colors based on the regioselectivity upon the substrate acceptors. (**B**) Putative substrate for OsUGT1-catalyzed glycosylation. GTs: glycosyltransferases.

Phylogenetic tree analysis is an effective means for functional prediction, enabling us to suggest a particular activity simply based on sequence information prior to exactly functional characterization of a new GT gene. Thirty GTs, including OsUGT1, were therefore used to build a phylogenetic tree that allowed the understanding of substrate specificities of OsUGT1. Firstly, these GTs were aligned by the Clustal X program. Subsequently, a neighbor-joining tree was derived from the multiple sequence alignment, using five sterol GTs as an outgroup. As shown in Figure 3A, these GTs are divided into two distinct branches. Five sterol GTs are clustered into one branch, while other flavonoid GTs are clustered into another branch. This divergence may reflect the substrate difference of GTs contained in the two branches. OsUGT1 is clustered into the same group with flavonoid GTs, suggesting OsUGT1 may be a flavonoid GT. This notion is consistent with the prediction result of CD-search program (Figure S2). The flavonoid GTs were further divided into three distinct clusters, 3-*O*-G, 5-*O*-G and 7-*O*-G clusters based on their regioselectivity (Figure 3A). 3-*O*-G, 5-*O*-G and 7-*O*-G groups include the enzymes preferably catalyzing glycosylation at the C-3, C-5 and C-7 positions of flavonoids, respectively. OsUGT1 is clustered to 7-*O*-G group, suggesting that OsUGT1 may attack the hydroxyl group at C-7 position of flavonoids. On the other hand, OsUGT1 is clustered as the same phylogenetic group with a putative 5,6-dihydroxyindoline-2-carboxylic acid (*cyclo*-DOPA) 5-*O*-glucosyltransferase (KP174811.1), suggesting OsUGT1 may act on the flavonoids showing similar structure with *cyclo*-DOPA [26]. *Cyclo*-DOPA has a bicyclic structure consisting of a six-membered benzene ring fused to a five-membered nitrogen-containing ring (Figure 3B). In flavonoids, aurones have a skeleton similar to *cyclo*-DOPA (Figure 3B). Cumulatively, we inferred that OsUGT1 was likely to be able to glycosylate aurones. Thus, the representative aurone sulfuretin (1) was selected as the substrate to incubate with the purified OsUGT1 for exactly functional characterization.

### 3.4. Heterologous Expression and Purification of OsUGT1

As shown in SDS-PAGE, under the induction of IPTG, an intense band with an apparent molecular mass of 54 kDa was present in the crude extract of *Trans*etta (DE3) [pET28aOsUGT1] but not in that of the control strain *Trans*etta (DE3) [pET-28a (+)] (Figure S3A), suggesting OsUGT1 was successfully expressed in *Escherichia coli* in a soluble form (Figure S3A). Also, the soluble expression of OsUGT1 was confirmed by western-blotting analysis. As shown in Figure S3B, a specific band was visualized in the crude extract of *Trans*etta (DE3) [pET28aOsUGT1] after incubation with an anti-His-tag antibody. On the contrary, no any bands appeared in the control lysis of *Trans*etta (DE3) [pET-28a (+)]. Cumulatively, bacteria-derived OsUGT1 was successfully obtained in a soluble form (Figure S3). Subsequently, the hexahistidine-tagged OsUGT1 was purified by Ni^2+^ affinity chromatography to near homogeneity (Figure S3). The concentration of the purified OsUGT1 was determined to 9.55 mg/mL.

### 3.5. OsUGT1 is an Aurone Glycosyltransferase

**Figure 4:**
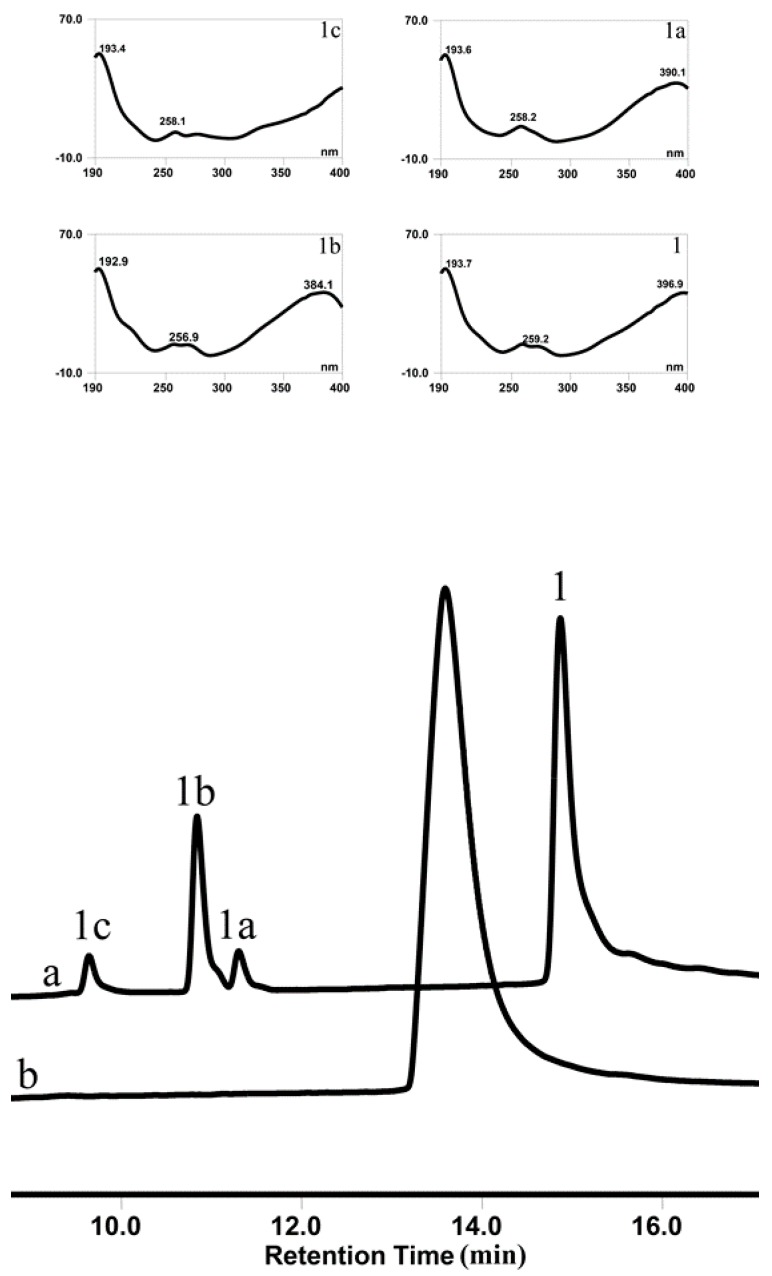
HPLC chromatogram of reaction product of sulfuretin (**1**) incubated with OsUGT1 (**a**) or without OsUGT1 (**b**). UV spectra of **1** and three enzymatic products **1a**, **1b** and **1c** are shown in upper panels.

To verify this speculation that OsUGT1 may be an aurone glycosyltransferase, a representative aurone sulfuretin (1) was used as the sugar acceptor to react with the sugar donor UDP-Glc in the presence of the purified OsUGT1. As shown in Figure 4, the enzyme assay using purified OsUGT1 with sulfuretin (1) and UDP-Glc yielded three peaks, 1a, 1b and 1c. On the contrary, the control reaction without OsUGT1 did not generate any new peaks, suggesting the three peaks were OsUGT1-assisted metabolites (Figure 4). The ultraviolet (UV) spectra of these three products were the same as that of sulfuretin (1), indicating that they had similar structural skeleton with that of sulfuretin (1) (Figure 4). These metabolites were collected and then subjected to mass spectrometry (MS) analysis. As shown in Figure S4, these metabolites shared the molecular ion [M+H]^+^ of *m/z* 433.3, 433.1 and 433.3, respectively, corresponding to the monoglycosylated metabolites of sulfuretin (1). To further clarify the exact structures of these metabolites, NMR analysis was carried out (Figures S5–S10). The ^1^H- and ^13^C-NMR data of these glycosylated sulfuretin were tabulated in Table 1 and Table 2. The *β*-configuration of sugars were concluded from the anomeric proton signals at *δ* 4.82 (1H, d, 7.4 Hz, H-1″), 4.84 (1H, d, 7.4 Hz, H-1″) and 5.16 (1H, d, 7.4 Hz, H-1″) in the 1H NMR spectrum [27]. The glucosyl moiety from the significantly downshifted signal of C-3′ (*δ* 145.6) and heteronuclear multiple bond correlation (HMBC) between H-1″ and C-3′ (Figure S6), suggested that the *O*-glucose moiety was attached to C-3′. In the HMBC spectrum of compound 1b, long-range correlations between H-1″ and C-4′ (*δ* 147.4), demonstrated that the glucosyl group was located at C-4′ (Figure S8). The long-range heteronuclear correlations of H-1″/C-6 of the HMBC spectrum of 1c (Figure S10) further confirmed *O*-glucose moiety was attached to C-3′ in 1c. Thus, the three compounds 1a, 1b and 1c were thus assigned as sulfuretin 3′-glucoside (1a), 4′-glucoside (1b) and 6-glucoside (1c), respectively. To the best of our knowledge, of the three newly formed sulfuretin glucosides, sulfuretin 4′-glucoside (1b) is a new compound [1]. These data indicated that OsUGT1 had a broad regioselectivity, attaching sugar moiety to the hydroxyl group at C-3′, C-4′ or C-6 position of sulfuretin (1). Overall, OsUGT1 was identified exactly to be an aurone glycosyltransferase, converting sulfuretin (1) to form three monoglycosides (Figure 1).

### 3.6. Substrate Specificity of OsUGT1-Catalyzed Glycosylation

Besides sulfuretin (1), other compounds listed in Figure 2 and Table S1 were used as the sugar acceptors to react with OsUGT1 and UDP-Glc for the examination of acceptor specificity. OsUGT1-containing crude extract was used as a biocatalyst for glycosylation reaction due to its simple preparation. Each member of 41 compounds was incubated with UDP-Glc in the presence of 20 μL OsUGT1-containing crude extract (derived from 1 mL cell). Of the 41 compounds, only 6 flavonoids, including luteolin (2) (Figures S11 and S12), 7,8-dihydroxyflavone (3) (Figures S13 and S14), 5,7-dihydroxyflavone (4) (Figures S15 and S16), 6-hydroxyflavone (5) (Figures S17 and S18) and 3,2′-dihydroxyflavone (6) (Figures S19 and S20), were found to undergo measurable glucosylation with UDP-Glc. On the other hand, OsUGT1 displayed no observable activity towards other 35 compounds including 5-hydroxyflavone (7), anthraquinones (8), steroids (9–25), terpenoids (26–28), phenolic acids (29–33) and alkaloids (34–41). These data collectedly indicated OsUGT1 was a flavonoid glycosyltransferase specific for flavonoid acceptors.

Under the action of OcUGT1, luteolin (2) was glycosylated to form three glucosides (2a–2c). Mass spectrum analyses and their co-elution with standard on reverse HPLC revealed that these three products were luteolin 3′-glucoside (2a), 4′-glucoside (2b) and 7-glucoside (2c), respectively (Figure S12). This result suggested that OsUGT1 preferred to attack the hydroxyl groups at C-3′, C-4′ and C-7 positions but not C-5 position. This notion was further confirmed by the glucosylation results of 5,7-dihydroxyflavone (4) (Figures S15 and S16) and 5-hydroxyflavone (7). After incubated with the purified OsUGT1, 5,7-dihydroxyflavone (4) was glucosylated to form one glucosylated product, 5,7-dihydroxyflavone 7-*O*-glucoside (4a). On the other hand, the incubation of 5-hydroxyflavone (7) with the purified OsUGT1 resulted in no any glucosylated products. Both indicated that OsUGT1 was able to attach sugar moiety to C-7 position but not C-5 position of flavones.

Also, OsUGT1 was able to glycosylate 3,2′-dihydroxyflavone (6) to form two corresponding monoglucosides (Figures S19 and S20). In addition, OsUGT1-catalyzed glucosylation of 6-hydroxyflavone (5) was observable (Figures S17 and S18). As shown in Figures S13 and S14, after incubated with OsUGT1, 7,8-dihydroxyflavone (3) was glucosylated to yield a major product of 7-*O*-monoglucoside (Figures S13 and S14). Hence, OsUGT1 was determined to be a flavonoid glycosyltransferase with broad regio-specificity, attaching sugars to C-2′, 3′, 4′, 3, 6 and 7 positions of flavonoids.

The sugar donor specificity of OsUGT1 was also investigated. Of the four donor substrates tested, including UDP-Glc, UDP-GlcNAc, UDP-Xyl and UDP-Ara, only UDP-Glc was able to support the glucosylation of sulfuretin (1). Cumulatively, OsUGT1 was determined to be an aurone flavonoid glucosyltransferase, consistent with the predicting results of CD-search and phylogenetic tree analysis.

### 3.7. Enzymatic Properties of OsUGT1-Catalyzed Glycosylation

**Figure 5:**
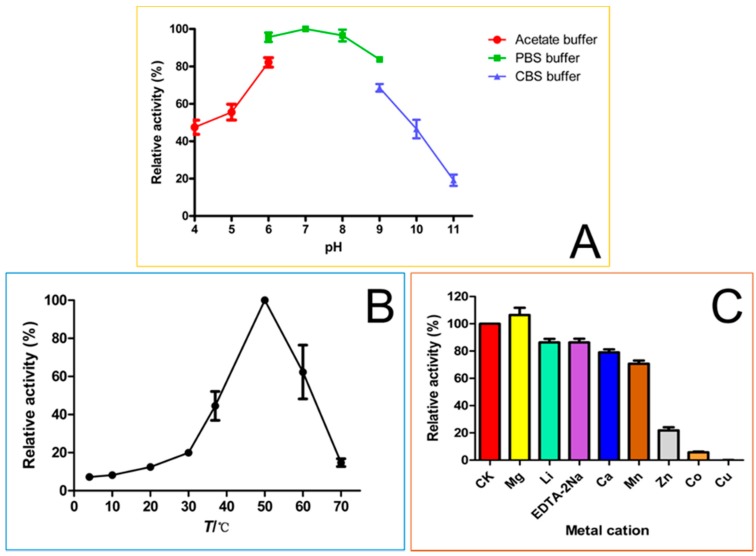
The effects of pH (**A**), temperature (**B**) and cation ions (**C**) on the glucosylation activity of OsUGT1.

Because of the low availability of sulfuretin (1), we chose another flavonoid substrate 7,8-dihydroxyflavone (3) for the enzymatic characterization of OsUGT1-catalyzed glycosylation. The enzymatic activity of OsUGT1 towards 7,8-dihydroxyflavone (3) was tested over a pH range of 4.0–11.0. The pH optimum was observed to fall around 7.0. The activity decreased significantly when pH exceeded 8.0. The residual activity of OsUGT1 was 20% when pH was 11.0 (Figure 5A).

OsUGT1 showed a broad temperature tolerance, exhibiting activity form 0–70 °C. The optimal OsUGT1 activity took place at 50 °C. When the temperature exceeded 50 °C, the glycosylation activity of OsUGT1 dropped markedly (Figure 5B).

The effect of various metal cations and chelating reagent on OsUGT1 activity was also tested (Figure 5C). The addition of 1 mM Mg^2+^ had a slightly stimulating effect on the OsUGT1-catalyzed glycosylation of 7,8-dihydroxyflavone (3). On the contrary, OsUGT1 activity was inhibited by the addition of Li^+^, EDTA-2Na, Ca^2+^, Mn^2+^, Zn^2+^, Co^2+^ and Cu^2+^ at the same concentration. Specifically, the metal ions Li^+^, Ca^2+^, Mn^2+^ and the chelating reagent EDTA-2Na had a slightly inhibitory effect upon OsUGT1 activity, whereas Zn^2+^, Co^2+^ and Cu^2+^ displayed significantly inhibitory effect on OsUGT1 activity. As shown in Figure 5C, the addition of Cu^2+^ caused almost complete loss of OsUGT1 activity.

The basic kinetic parameters for 7,8-dihydroxyflavone (3) and sulfuretin (1), *K*_m_ and *V*_max_, were determined from Lineweave-Burk equation. The detailed kinetic parameters were summarized in Table 3. As shown in Table 3, the *K*_m_ value of sulfuretin (1) is lower than that of 7,8-dihydroxyflavone (3), indicating OsUGT1 displayed higher affinity towards sulfuretin (1) (Table 3). 

### 3.8. OsUGT1-Catalyzed Transglucosylation Action

**Figure 6:**
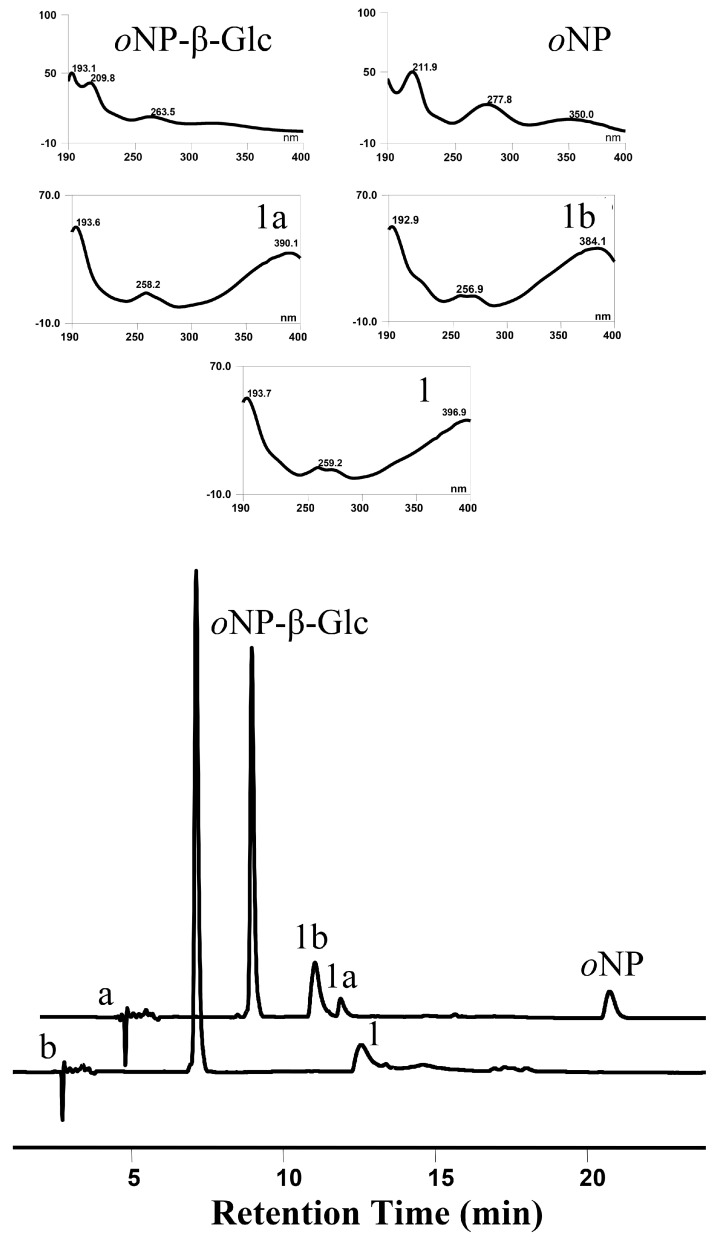
HPLC chromatogram of transglucosylation reaction sulfuretin (1) and *o*NP-β-Glc mediated by OsUGT1 (a) or no OsUGT1 (b). UV spectra of 1, 1a, 1b, *o*NP-β-Glc and *o*NP are shown in upper panels. *o*NP-β-Glc: *o*-nitrophenyl-β-d-glucopyranoside; *o*NP: *o*-nitrophenol.

In this study, the glycosidase activities of OsUGT1, namely transglycosylation and hydrolysis activities, were also investigated. The transglycosylation capability of OsUGT1 on sulfuretin (1) and *o*NP-β-Glc was firstly explored. As shown in Figure 6, when sulfuretin (1) was incubated with *o*NP-β-Glc, an intermolecular transglucosylation occurred between sulfuretin (1) and *o*NP-β-Glc. Specifically, the glucosyl moiety was transferred from *o*NP-β-Glc to sulfuretin (1), thereby forming two monoglucosides 1a and 1b. Meanwhile, *o*NP-β-Glc was deglucosylated to yield *o*NP (Figure 6). As we all know, GT-assisted glycosylation of small molecules was hampered essentially by the requirement of expensive nucleotide diphospho-sugars (NDP-sugars) as the donor [28]. It is therefore important to isolate glycosyltransferases capable of utilizing cheap alternatives of NDP-sugars for glycosylation of small molecules. Aryl-substituted glycosides like *o*NP-β-Glc and *o*NP-β-Gal are cheap sugar donors and usually applied for the biosynthesis of new glycosides via transglycosylation [29,30]. In this investigation, we found that OsUGT1 was able to transfer glucosyl group from *o*NP-β-Glc to sulfuretin (**1**) to form sulfuretin glucosides (Figure 1 and Figure 6). These data revealed that OsUGT1-catalyzed biosynthesis of sulfuretin glucosides was achieved via glucosylation or transglucosylation reaction, which no doubt broadened the application of OsUGT1 in the biosynthesis of sulfuretin glucosides.

### 3.9. OsUGT1-Catalyzed Hydrolysis

**Figure 7:**
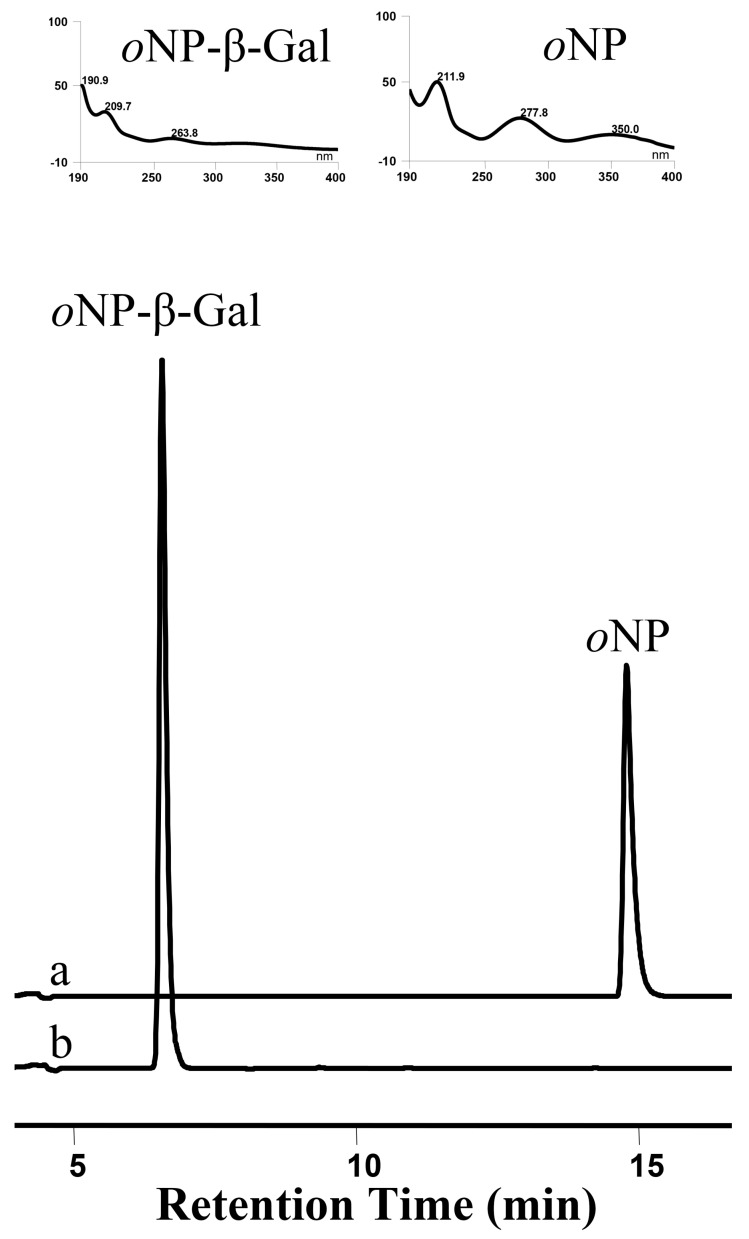
HPLC chromatogram of hydrolysis reaction of *o*NP-β-Gal in the presence (a) or absence (b) OsUGT1. UV spectra of *o*NP-β-Gal and *o*NP are shown in upper panels. *o*NP-β-Gal: *o*-nitrophenyl-β-d-galactopyranoside.

*o*NP-β-Gal was used as the substrate to test the hydrolysis activity of OsUGT1. After incubation of *o*NP-β-Gal with 10 μg purified OsUGT1 at 37 °C for 2 h, the glycosidic bond hydrolysis of *o*NP-β-Gal was observable (Figure 7). The hydrolytic substrate *o*NP-β-Gal was completely hydrolyzed and a new peak representing hydrolysis product *o*NP was thus detected in the reaction mixture, as shown in Figure 7. These data indicated that OsUGT1 had a hydrolysis activity. These evidences revealed that OsUGT1 has transglucosylation and hydrolysis activities, indicating OsUGT1 is a glycosidase. We had previously isolated a GT designated as OcUGT1 with glycosidase activity from *Ornithogalum caudatum* [16]. Although OcUGT1 and OsUGT1 were from different species, they displayed similar functions. For example, the two enzymes were able to glycosylate flavonoids. Moreover, both of the two glycosyltransferases could hydrolyze *o*NP-β-Gal to form *o*NP-β. These data suggested that glycosyltransferases with glucosidase activity might be widespread in plants. Although OcUGT1-catalyzed glucosylation towards flavonoids were demonstrated, there were no evidences that OcUGT1 was able to glycosylate aurones [16]. Thus, OsUGT1 is regarded as the first aurone glycosyltransferase with glycosidase action. Obviously, this multifunctionality of OcUGT1 will broaden the application of OcUGT1 in glucosylations of aurones.

## 4. Conclusions

An aurone glycosyltransferase, designated as OsUGT1, was isolated from *O. saundersiae* and then demonstrated to be active towards sulfuretin and other flavonoids. Moreover, OsUGT1 was determined to exhibit glycosidase activity, catalyzing the generation of sulfuretin glucosides from *o*NP-β-Glc via intermolecular transglucosylation. Cumulatively, herein reported an aurone glycosyltransferase with glycosidase activity for the first time.

## Figures and Tables

**Table 1:** ^1^H NMR (600 MHz) spectroscopic data (*δ* in ppm, *J* in Hz) for compounds **1a**~**1c** in DMSO-*d*_6_.

Position	1a	1b	1c
4	7.60, d (8.5)	7.62, d (8.5)	7.70, d (8.5)
5	6.71, m	6.73, dd (8.5, 1.9)	6.92, dd (8.5, 2.0)
7	6.76, d (1.9)	6.80, d (1.9)	7.14, d (2.0)
10	6.71, m	6.69, s	6.71, s
2′	7.74, d (1.9)	7.49, d (2.0)	7.44, d (2.0)
5′	6.94, d (8.3)	7.20, d (8.6)	6.86, d (8.2)
6′	7.51, dd (8.3, 1.9)	7.37, dd (8.6, 2.0)	7.31, dd (8.2, 2.0)
	Glc	Glc	Glc
1″ 1‴	4.82, d (7.4)	4.84, d (7.4)	5.16, d (7.4)
H of sugar	3.0–3.8	3.0–3.8	3.0–3.8

DMSO: dimethyl sulfoxide.

**Table 2:** ^13^C NMR (150 MHz) spectroscopic data (*δ* in ppm) for compounds **1a**~**1c** in DMSO-*d*_6_.

Position	1a	1b	1c
2	145.9, C	146.9, C	145.9, C
3	181.3, C	181.8, C	182.0, C
4	125.8, CH	126.3, CH	125.8, CH
5	113.0, CH	113.4, CH	114.0, CH
6	166.4, C	166.9, C	165.1, C
7	98.6, CH	99.0, CH	99.8, CH
8	167.7, C	168.2, C	167.5, C
9	113.0, C	113.5, C	115.8, C
10	111.5, CH	111.4, CH	113.4, CH
1′	127.2, C	126.9, C	125.2, C
2′	118.7, CH	118.4, CH	118.8, CH
3′	145.6, C	147.2, C	146.1, C
4′	148.8, C	147.4, C	148.8, C
5′	116.5, CH	116.5, CH	116.5, CH
6′	123.7, CH	124.3, CH	123.7, CH
	Glc	Glc	Glc
1″	101.9, CH	101.8, CH	100.3, CH
2″	73.3, CH	73.7, CH	73.6, CH
3″	76.0, CH	76.3, CH	76.8, CH
4″	69.6, CH	70.3, CH	70.0, CH
5″	77.1, CH	77.7, CH	77.6, CH
6″	60.6, CH_2_	61.2, CH_2_	61.1, CH_2_

**Table 3:** Kinetic parameters of recombinant OsUGT1.

Substrate	*K*_m_ (mM)	*V*_max_ (mM/h)
sulfuretin	0.159 ± 0.011	0.208 ± 0.004
7,8-dihydroxyflavone	0.713 ± 0.069	1.695 ± 0.085

*K*_m_: Michaelis constant; *V*_max_: maximum velocity.

## Supplementary Materials

The following are available online at http://www.mdpi.com/2073-4425/9/7/327/s1; Figure S1: PCR product visualized on the agarose gel; Figure S2: List of conserved domain hits; Figure S3: Detection of the recombinant OsUGT1 by SDS-PAGE and Western-blotting; Figure S4: Mass spectra of metabolites 1a, 1b and 1c; Figure S5: ^1^H NMR spectrum and ^13^C NMR spectrum of 1a; Figure S6: HMBC spectrum of 1a; Figure S7: ^1^H NMR spectrum and ^13^C NMR spectrum of 1b; Figure S8: HMBC spectrum of 1b; Figure S9: ^1^H NMR spectrum and ^13^C NMR spectrum of 1c; Figure S10: HMBC spectrum of 1c; Figure S11: HPLC chromatogram of glucosylation of luteolin with OsUGT1 or without OsUGT1; Figure S12: The mass spectra of glucosylated metabolites 2a, 2b and 2c; Figure S13: HPLC chromatogram of glucosylation of 7,8-dihydroxyflavone with OsUGT1 or without OsUGT1; Figure S14: The mass spectrum of glucosylated metabolite 3a; Figure S15: HPLC chromatogram of glucosylation of 5,7-dihydroxyflavone with OsUGT1 or without OsUGT1; Figure S16: The mass spectrum of glucosylated metabolite 4a; Figure S17: HPLC chromatogram of glucosylation of 6-hydroxyflavone with OsUGT1 or without OsUGT1; Figure S18: The mass spectrum of glucosylated metabolite 5a; Figure S19: HPLC chromatogram of glucosylation of 3,2′-dihydroxyflavone with OsUGT1 or without OsUGT1; Figure S20: The mass spectra of glucosylated metabolites 6a and 6b; Table S1: Sugar acceptors used in this study; Table S2: The primers used in this study; Table S3: Plasmids and strains used in this research.
