# Supplementary material for: Transcriptome-Wide Identification of an Aurone Glycosyltransferase with Glycosidase Activity from Ornithogalum saundersiae
Source: Genes (Basel). 2018 Jun 28;9(7):327. doi: 10.3390/genes9070327 (PMC6071076; doi:10.3390/genes9070327)
Supplement: Supplementary file 1 [file genes-09-00327-s001.pdf]

Table captions

Table 1 Sugar acceptors used in this study

“√” means that the compound is able to react with OsUGT1; “×” indicates no reaction with OsUGT1

Table S2 The primers used in this study

Table S3 Plasmids and strains used in this research

Table S1

No	Sugar acceptor	CAS Number	Reaction
	Flavonoids		
1	Sulfuretin	120-05-8	√
2	Luteolin	491-70-3	√
3	7,8-Dihydroxyflavone	38183-03-8	√
4	5,7-Dihydroxyflavone	480-40-0	√
5	6-Hydroxyflavone	6665-83-4	√
6	3,2′-Dihydroxyflavone	6068-76-4	√
7	5-Hydroxyflavone	491-78-1	×
	Anthraquinones		
8	Rhein	478-43-3	×
	Steroids		
9	Dehydroepiandrosterone	53-43-0	×
10	17β-Estradiol	50-28-2	×
11	17α-Estradiol	57-91-0	×
12	Methyltestosterone	58-18-4	×
13	Testosterone	58-22-0	×
14	15β-Hydroxytestosterone	39605-73-7	×
15	2β-Hydroxytestosterone	10390-14-4	×
16	16β-Hydroxytestosterone	17528-90-4	×
17	16α-Hydroxytestosterone	63-01-4	×
18	Cortisone	53-06-5	×
19	Hydrocortisone	50-23-7	×
20	β-Ecdysone	5289-74-7	×
21	Cholesterol	57-88-5	×
22	β-Sitosterol	83-46-5	×
23	Ergosterol	57-87-4	×
24	Diosgenin	512-04-9	×
25	Cholic acid	81-25-4	×
	Terpenoids		
26	Protopanaxatriol	1453-93-6	×
27	Oleanolic acid	508-02-1	×
28	Ursolic acid	77-52-1	×
	Phenolic acids		
29	Caffeic acid	331-39-5	×
30	Ferulic acid	1135-24-6	×
31	*p*-Coumaric acid	501-98-4	×
32	Sinapic acid	530-59-6	×
33	DL-4-Hydroxymandelic acid	1198-84-1	×
	Alkaloids		
34	2,6-Dihydroxyquinoline	19315-93-6	×
35	Higenamine	5843-65-2	×

36	5-Hydroxyindole acetic acid	54-16-0	×
37	Berberine	2086-83-1	×
38	Camptothecin	7689-03-4	×
39	Securinine	5610-40-2	×
40	Matrine	519-02-8	×
41	Peimisine	19773-24-1	×

Table S2

Primer	Sequence (5'to 3')	Description
Fcomp28733-1	CAATCGTCTCCTCTTGGAC	Forward primer used for *OsUGT1* amplification in the first round
Rcomp28733-1	CATCAACTGATTGCTATGTC	Reverse primer used for *OsUGT1* amplification in the first round
Fcomp28733-2	ATGGAAGGGAAAAAACAACAT	Forward primer used for *OsUGT1*amplification in the second round
Rcomp28733-2	TTACACAAAAATCATGCAAAAG	Reverse primer used for *OsUGT1* amplification in the second round
F28a28733	CAAATGGGTCGCGGATCCGAATTCATGGAAGGGAAAAAACAA	Forward primer used for the construction of pET28aOsUGT1
R28a28733	GTGCTCGAGTGCGGCCGCAAGCTTTCAGGTGACAACCCTTTT	Reverse primer used for the construction of pET28aOsUGT1

Table S3

Strains/plasmids	Description	Source/Reference
Strain		
*Trans*1-T1	F^- φ80 (*lac*Z) ΔM15Δ*lac*X74 *hsd*R (r_k^-,m_k^+) Δ*rec*A1398*end*A1*ton*A	TransGen, Beijing, China
*Trans*etta (DE3)	F^-*omp*T*hsd*S_B($r_B^-m_B^-$)*gal dcm* (DE3)pRARE(argU, argW,ilex,glyT,leuW,proL)Cam^r)	TransGen, Beijing, China
Plasmid		
pEASY™-Blunt	General cloning vector, T7 promoter, f1 ori, Amp^r and Kan^r	TransGen, Beijing, China
pET-28a (+)	General expression vector, T7 promoter, f1 ori, Kan^r	Novagen, Madison, USA
pEASY-OsUGT1	*pEASY*™-Blunt derived vector containing *OsUGT1 gene*	This study
pET28a-OsUGT1	pET-28a (+) derived vector containing *OsUGT1 gene*	This study

Figure legend

Figure S1 PCR product visualized on the agrose gel

PCR product amplified by nested PCR with (1) or without cDNA template (2). M represents molecular marker indicated on the left margin with bp.

Figure S2 List of conserved domain hits, PLN02863 (**A**), COG1819 (**B**) and MGT (**C**), using OsUGT1 as the query sequence.

Figure S3 Detection of the recombinant OsUGT1 by SDS-PAGE (A) and Western-blotting (B) .

Lane M; protein molecular weight marker; lane 1, the crude extract of *Trans*etta (DE3) [pET28aOsUGT1] induced with IPTG; lane 2, the crude extract of *Trans*etta (DE3) [pET-28a(+)] induced with IPTG; lane 3, the purified OsUGT1 protein; lane 4, western-blotting analysis of the crude extract containing the recombinant OsUGT1; lane 5, western-blotting analysis of the crude extract containing no OsUGT1

Figure S4 Mass spectra of metabolites **1a (A), 1b (B)** and **1c (C).**

Figure S5 ^{1}H NMR spectrum (600 MHz, DMSO-d_6) (**A**) and ^{13}C NMR spectrum of **1a** (150 MHz, DMSO-d_6) (**B**)

Figure S6 HMBC spectrum of **1a**

Figure S7 ^{1}H NMR spectrum (600 MHz, DMSO-d_6) (**A**) and ^{13}C NMR spectrum of **1b** (150 MHz, DMSO-d_6) (**B**)

Figure S8 HMBC spectrum of **1b**

Figure S9 ^{1}H NMR spectrum (600 MHz, DMSO-d_6) (**A**) and ^{13}C NMR spectrum of **1c** (150 MHz, DMSO-d_6) (**B**)

Figure S10 HMBC spectrum of **1c**

Figure S11 HPLC chromatogram of glucosylation of luteolin (**2**) with OsUGT1 (a) or without OsUGT1 (b) (A). The UV absorption spectrum of glucosylated metabolites **2a**-**2c** is similar to that of **2**. All of them are marked in the upper panel.

Figure S12 The mass spectra of glucosylated metabolites **2a (A), 2b (B)** and **2c(C).**

Figure S13 HPLC chromatogram of glucosylation of 7,8-dihydroxyflavone (**3**) with OsUGT1 (a) or without OsUGT1 (b) (A). The UV absorption spectrum of glucosylated metabolite **3a** is similar to that of **3**. Both are marked in the upper panel.

Figure S14 The mass spectrum of glucosylated metabolite **3a**.

Figure S15 HPLC chromatogram of glucosylation of 5,7-dihydroxyflavone (**4**) with OsUGT1 (a) or without OsUGT1 (b) (A). The UV absorption spectrum of glucosylated metabolite **4a** is similar to that of **4**. Both are marked in the upper panel.

Figure S16 The mass spectrum of glucosylated metabolite **4a**.

Figure S17 HPLC chromatogram of glucosylation of 6-hydroxyflavone (**5**) with OsUGT1 (a) or without OsUGT1 (b) (A). The UV absorption spectrum of glucosylated metabolite **5a** is similar to that of **5**. Both are marked in the upper panel.

Figure S18 The mass spectrum of glucosylated metabolite **5a**.

Figure S19 HPLC chromatogram of glucosylation of 3,2′-dihydroxyflavone (**6**) with OsUGT1 (a) or without OsUGT1 (b) (A). The UV absorption spectrum of glucosylated metabolites **6a** and **6b** is similar to that of **6**. All of them are marked in the upper panel.

Figure S20 The mass spectra of glucosylated metabolites **6a (A)** and **6b (B)**.

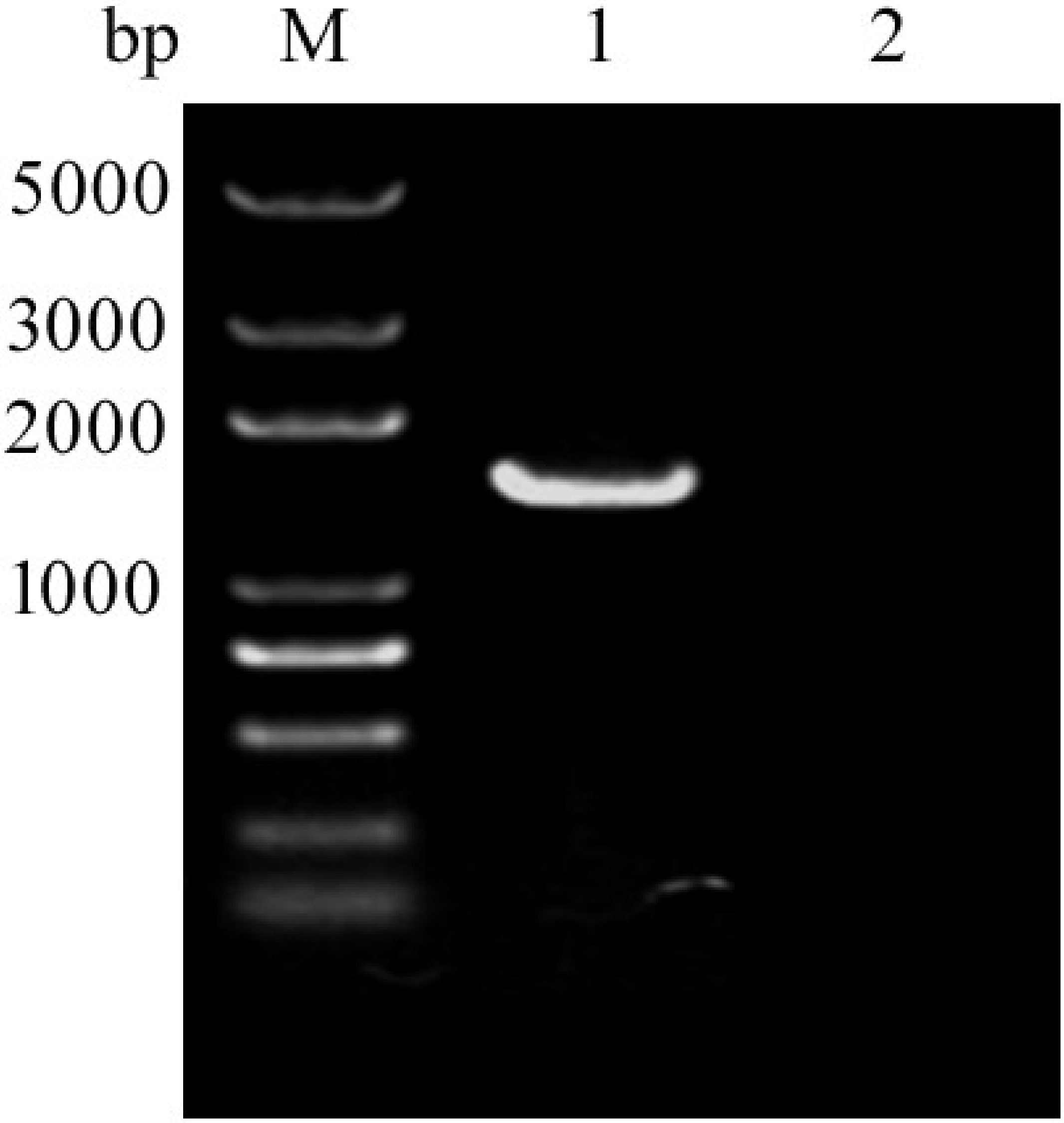

Figure S1

A

Pssm-ID: 215465 **Cd Length:** 477 **Bit Score:** 247.09 **E-value:** 1.78e-76

```
                         10        20        30        40        50        60        70        80
                 ....*....|....*....|....*....|....*....|....*....|....*....|....*....|....*....|
Query_52229    3 GKKQHVVLFPFMGQGHITPFLLLAEliHEYYPDYTITLVNTPLNIRNLQSSLPPGSKINLKSLPFDASShGLPPDTENTK 82
Cdd:PLN02863   7 PAGTHVLVFPFPAQGHMIPLLDLTH--RLALRGLTITVLVTPKNLPFLNPLLSKHPSIETLVLPFPSHP-SIPSGVENVK 83

                         90       100       110       120       130       140       150       160
                 ....*....|....*....|....*....|....*....|....*....|....*....|....*....|....*....|
Query_52229   83 AIPFHLFINLFRAsetLEPAFERLIAGITEEDGiPPLCIIADNFFSWTLHIARKFGVFHSTFLTSGAYGSALLFSLWKYL 162
Cdd:PLN02863  84 DLPPSGFPLMIHA---LGELYAPLLSWFRSHPS-PPVAIISDMFLGWTQNLACQLGIRRFVFSPSGAMALSIMYSLWREM 159

                        170       180       190       200       210       220       230       240
                 ....*....|....*....|....*....|....*....|....*....|....*....|....*....|....*....|
Query_52229  163 PHRNAYSDEFSLPDFPEI----RLHHTQLSNILVVADGGDAWSVLLRRLNELCTRSDAVLVNTVKEFEARGLSMLREQL- 237
Cdd:PLN02863 160 PTKINPDDQNEILSFSKIpncpKYPWWQISSLYRSYVEGDPAWEFIKDSFRANIASWGLVVNSFTELEGIYLEHLKKELg 239

                        250       260       270       280       290       300       310       320
                 ....*....|....*....|....*....|....*....|....*....|....*....|....*....|....*....|
Query_52229  238 QGPIFPVGPLLCTSASHSMGM----------DLSDWLDSQPSASVLYVSFGSQNTIRASQMLKLAMALEATSRPFIWVI 306
Cdd:PLN02863 240 HDRVWAVGPILPLSGEKSGLMerggpssvsvdDVMTWLDTCEDHKVVYVCFGSQVVLTKEQMEALASGLEKSGVHFIWCV 319

                        330       340       350       360       370       380       390       400
                 ....*....|....*....|....*....|....*....|....*....|....*....|....*....|....*....|
Query_52229  307 RPPVGFDVrvqfKSEWLPEGFEDRMRGtkQGFIVHQWAPQVEILSHPSTGAFLSHCGWNSVLESLVHGVPIIGWPLSAEQ 386
Cdd:PLN02863 320 KEPVNEES----DYSNIPSGFEDRVAG--RGLVIRGWAPQVAILSHRAVGAFLTHCGWNSVLEGLVAGVPMLAWPMAADQ 393
                                                                     PSPG motif

                        410       420       430       440       450       460       470       480
                 ....*....|....*....|....*....|....*....|....*....|....*....|....*....|....*....|
Query_52229  387 FYNSKFLMEEVGVCVEAARGNRESSEvkKEEVERAI-EIVMDNEEIRRKAKETARVLKSALKEEenntnvGSSLRGLKEF 465
Cdd:PLN02863 394 FVNASLLVDELKVAVRVCEGADTVPD--SDELARVFmESVSENQVERERAKELRRAALDAIKER------GSSVKDLDGF 465

                 ..
Query_52229  466 FQ 467
Cdd:PLN02863 466 VK 467
```

B

Pssm-ID: 224732 **Cd Length:** 406 **Bit Score:** 78.63 **E-value:** 1.05e-15

```
                       10        20        30        40        50        60        70        80
               ....*....|....*....|....*....|....*....|....*....|....*....|....*....|....*....|
Query_52229 272 VLYVSFGsqnTIRASQMLkLAMALEATSrpfiwvirppvGFDVRVQFksewLPEGFEDRMRGTKQGFIVHQWAPQVEILs 351
Cdd:COG1819 239 IVYVSLG---TVGNAVEL-LAIVLEALA-----------DLDVRVIV----SLGGARDTLVNVPDNVIVADYVPQLELL- 298

                       90       100       110       120       130       140       150       160
               ....*....|....*....|....*....|....*....|....*....|....*....|....*....|....*....|
Query_52229 352 hPSTGAFLSHCGWNSVLESLVHGVPIIGWPLSAEQFYNSKFLmEEVGVCVeAARGNRESSEVKKEEVERaieiVMDNEEI 431
Cdd:COG1819 299 -PRADAVIHHGGAGTTSEALYAGVPLVVIPDGADQPLNAERV-EELGAGI-ALPFEELTEERLRAAVNE----VLADDSY 371

                      170
               ....*....|....
Query_52229 432 RRKAKETARVLKSA 445
Cdd:COG1819 372 RRAAERLAEEFKEE 385
```

C

Pssm-ID: 273616 **Cd Length:** 392 **Bit Score:** 76.65 **E-value:** 4.41e-15

```
                          10        20        30        40        50        60        70        80
                  ....*....|....*....|....*....|....*....|....*....|....*....|....*....|....*....|
Query_52229   338 FIVHQWAPQVEILSHPStgAFLSHCGWNSVLESLVHGVPIIGWPLSAEQFYNSKFLmEEVGVCVEAARGnressEVKKEE 417
Cdd:TIGR01426 277 VEVRQWVPQLEILKKAD--AFITHGGMNSTMEALFNGVPMVAVPQGADQPMTARRI-AELGLGRHLPPE-----EVTAEK 348

                          90       100
                  ....*....|....*....|....*...
Query_52229   418 VERAIEIVMDNEEIRRKAKETARVLKSA 445
Cdd:TIGR01426 349 LREAVLAVLSDPRYAERLRKMRAEIREA 376
```

Figure S2

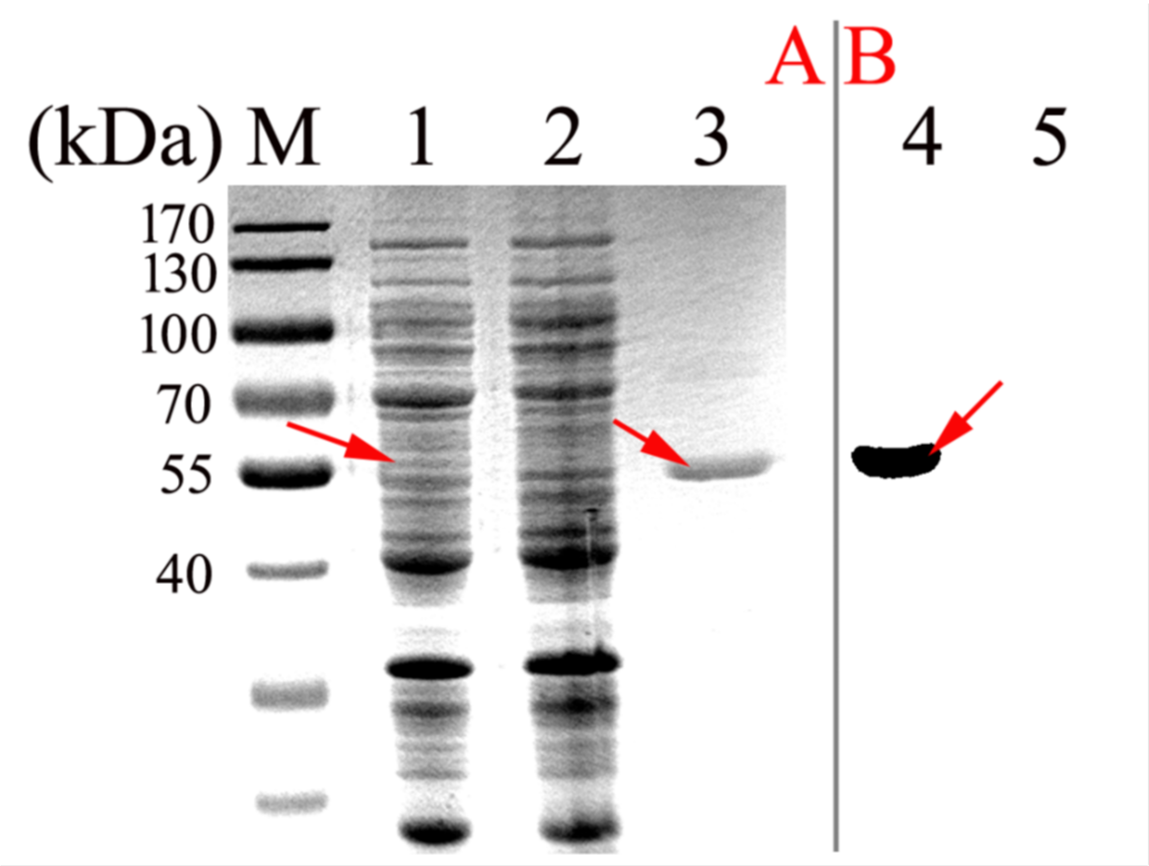

Figure S3

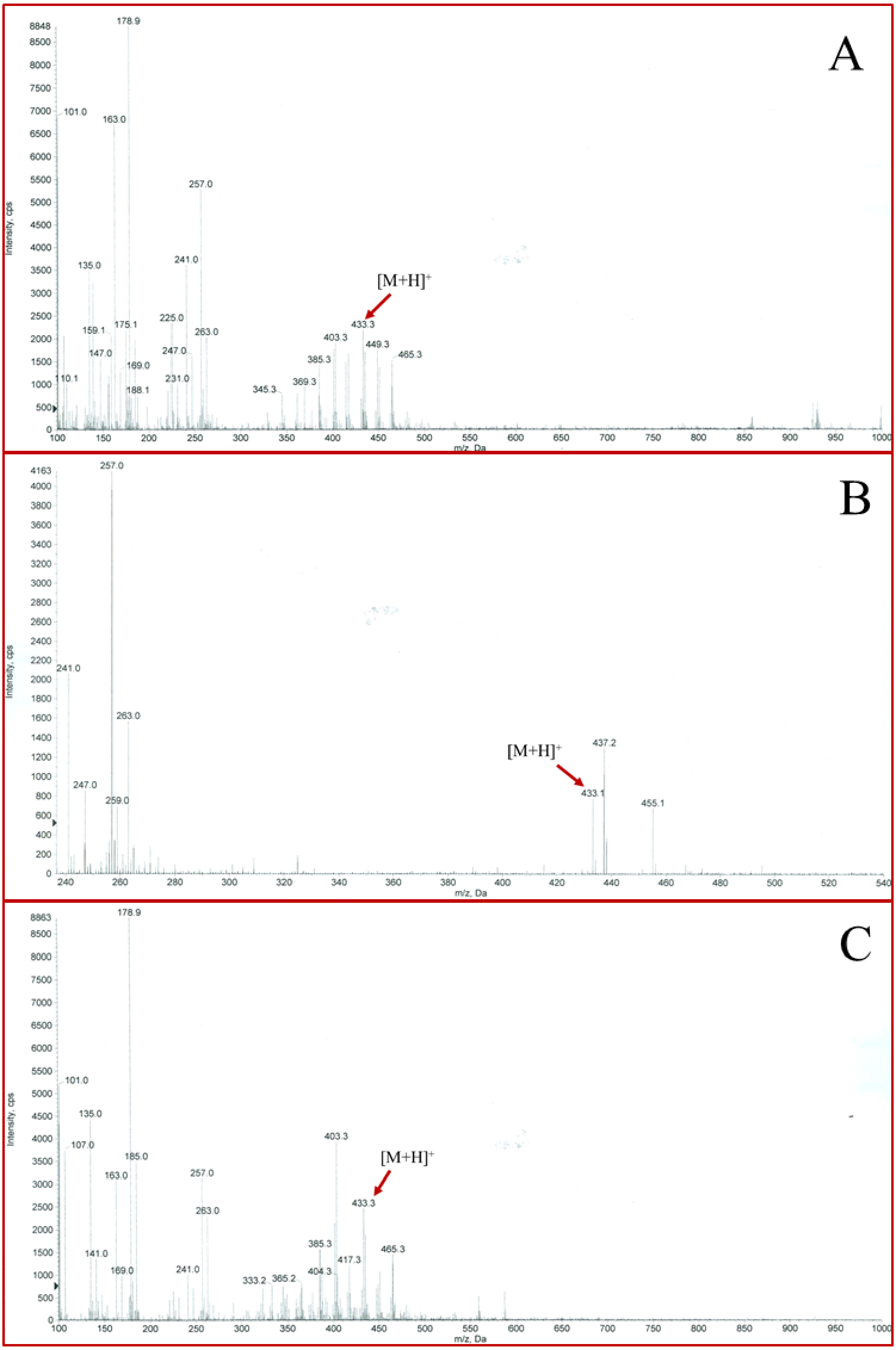

Figure S4

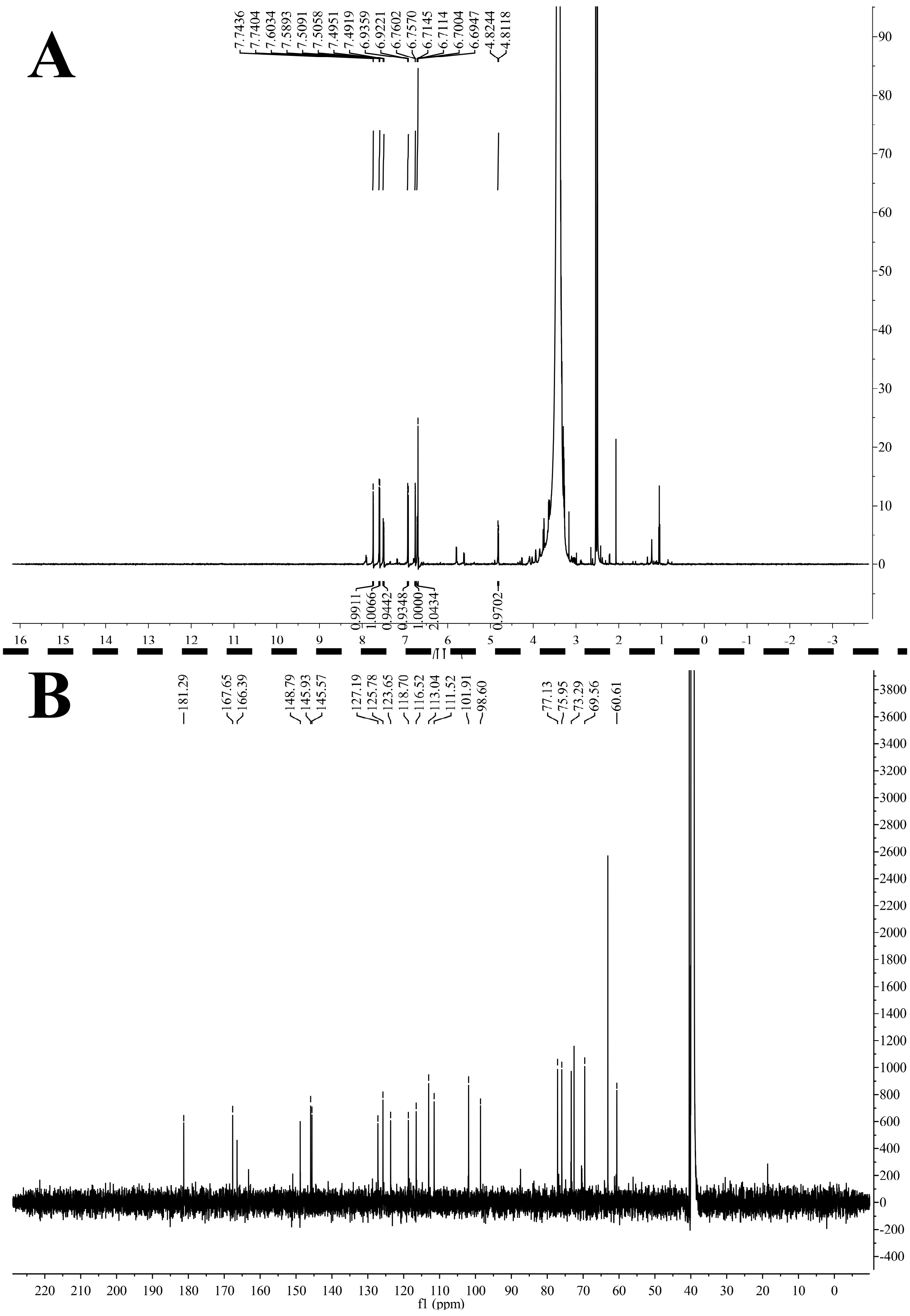
A
7.7436 7.7404 7.6034 7.5893 7.5091 7.5058 7.4951 7.4919 6.9359 6.9221 6.7602 6.7570 6.7145 6.7114 6.7004 6.6947 4.8244 4.8118
0.9911 1.0066 0.9442 0.9348 1.0000 2.0434 0.9702
B
181.29 167.65 166.39 148.79 145.93 145.57 127.19 125.78 123.65 118.70 116.52 113.04 111.52 101.91 98.60 77.13 75.95 73.29 69.56 60.61
f1 (ppm)

Figure S5

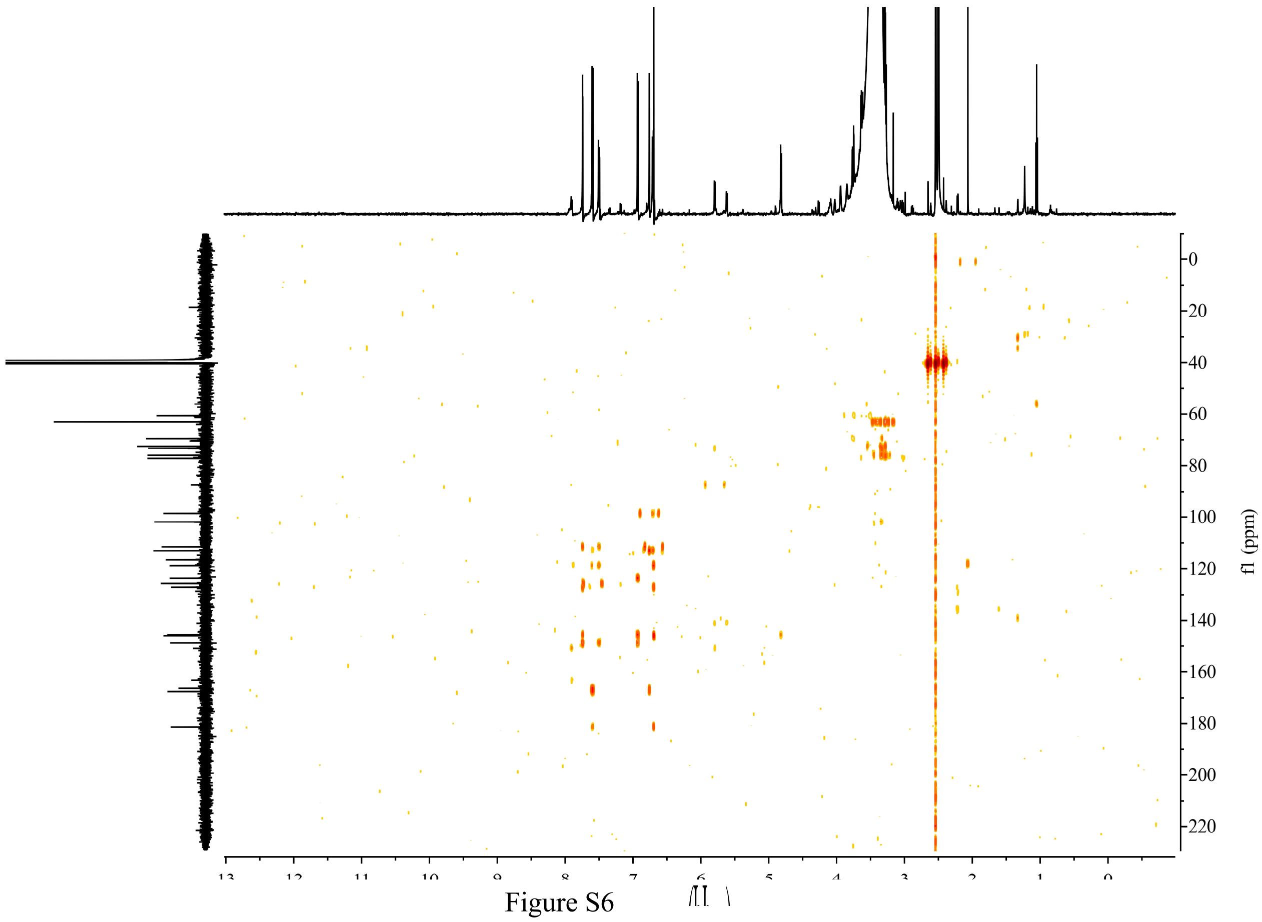

Figure S6

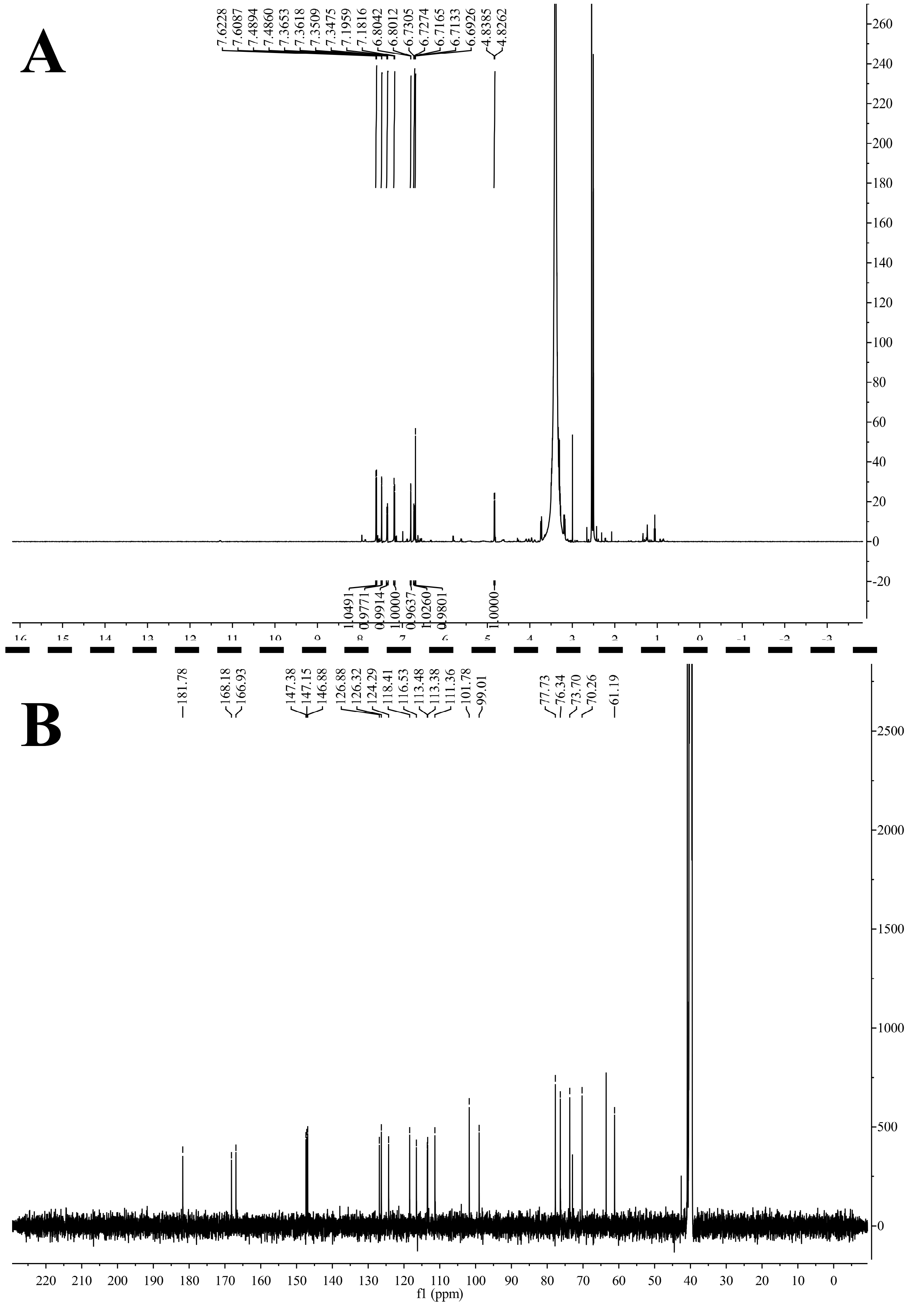

Figure S7

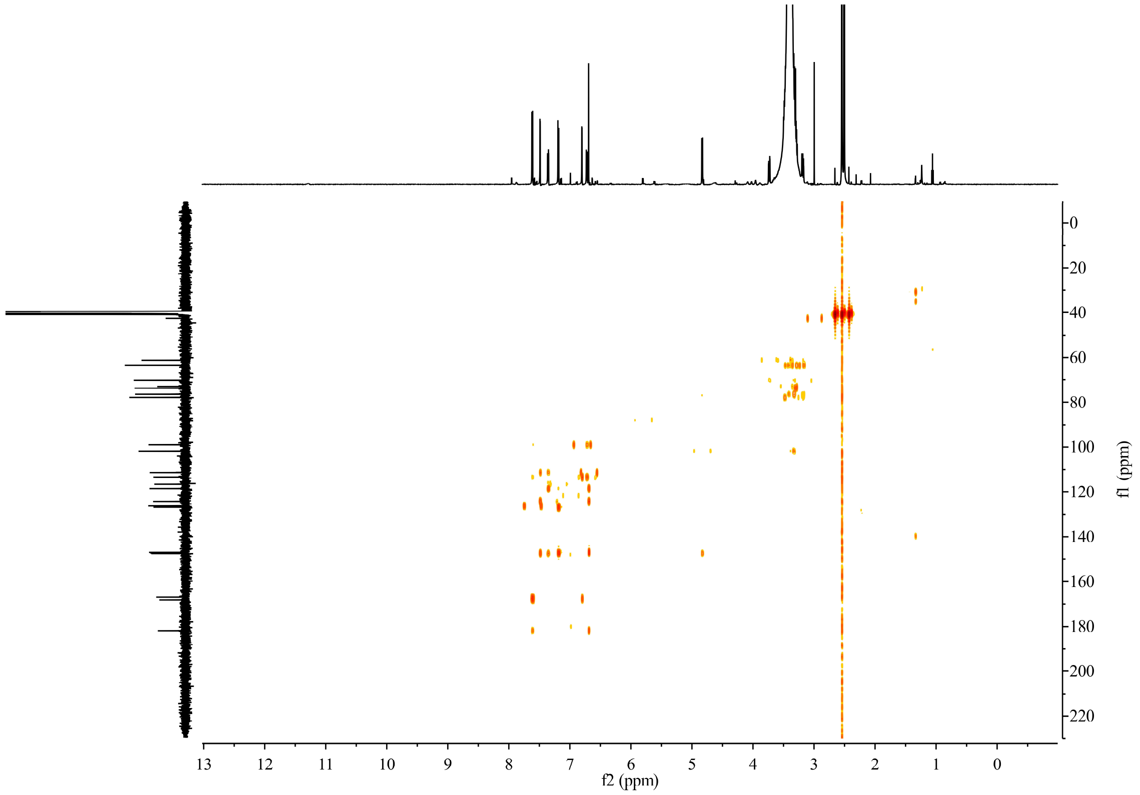

0
20
40
60
80
100
120
140
160
180
200
220
f1 (ppm)
13
12
11
10
9
8
7
6
5
4
3
2
1
0
f2 (ppm)

Figure S8

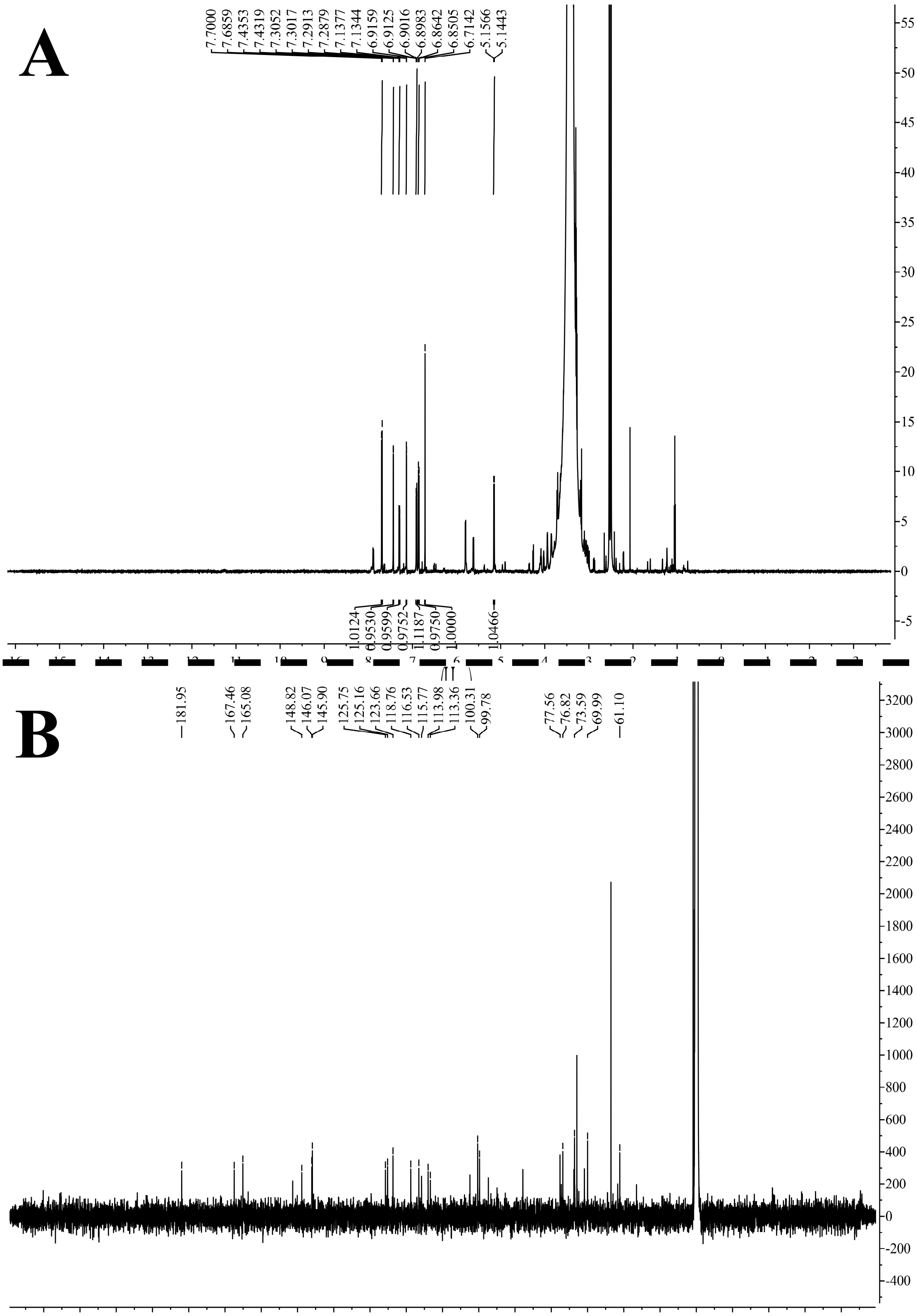

Figure S9

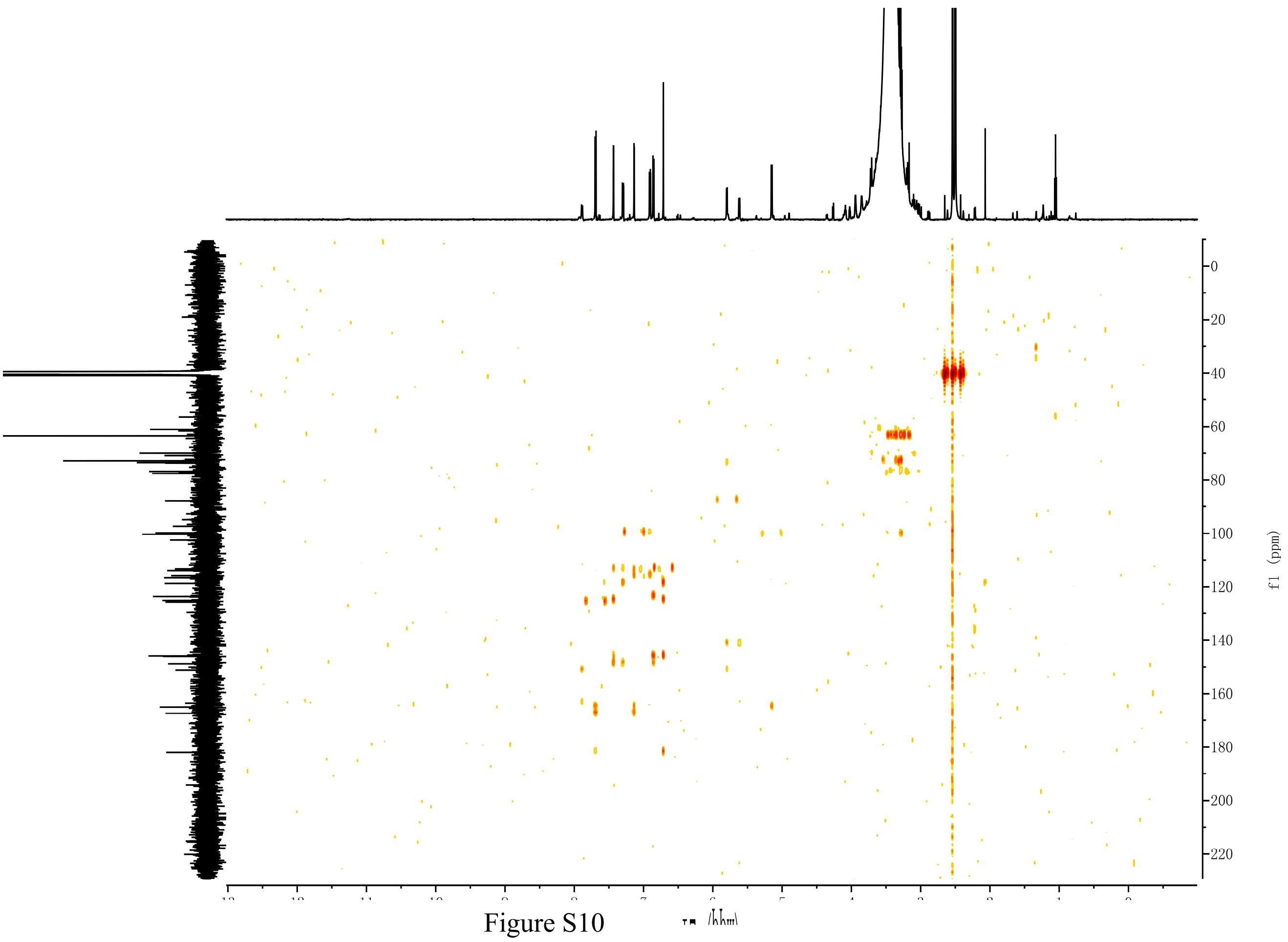

Figure S10

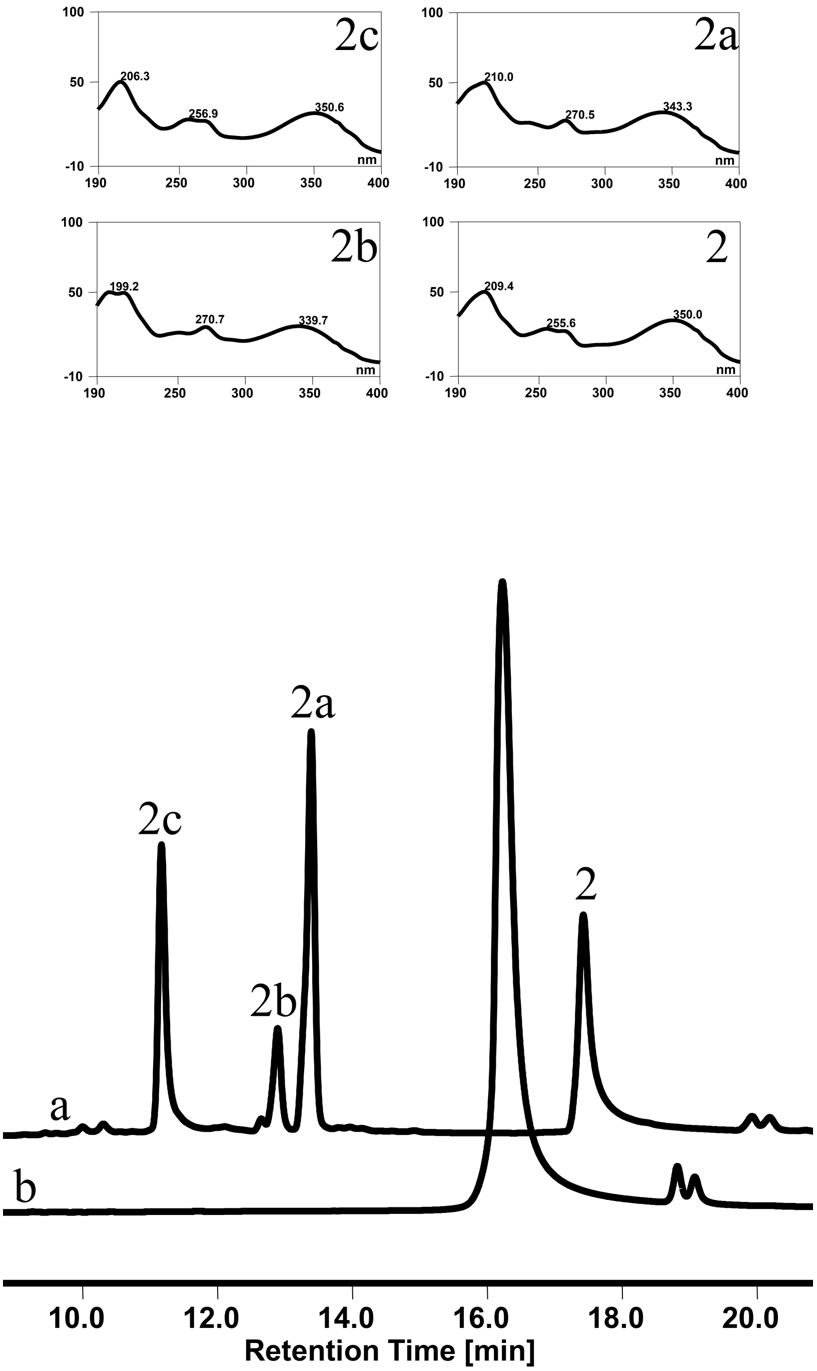

2c
206.3
256.9
350.6
2a
210.0
270.5
343.3
2b
199.2
270.7
339.7
2
209.4
255.6
350.0
100
50
-10
nm
190
250
300
350
400
a
b
10.0
12.0
14.0
16.0
18.0
20.0
Retention Time [min]

Figure S11

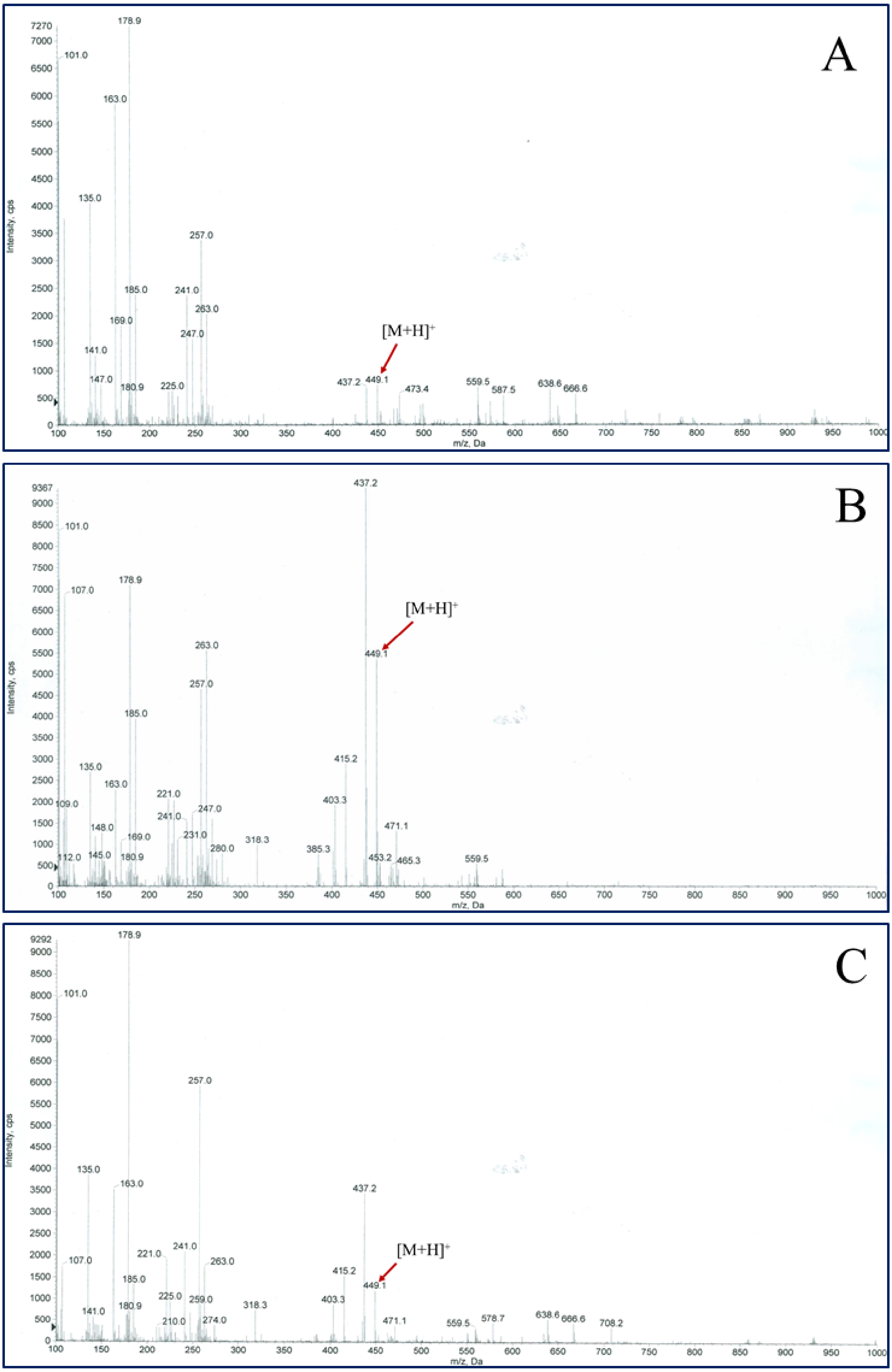
A
B
C
[M+H]+
Intensity, cps
m/z, Da
178.9
101.0
163.0
135.0
257.0
185.0
241.0
263.0
169.0
247.0
141.0
147.0
180.9
225.0
437.2
449.1
473.4
559.5
587.5
638.6
666.6
107.0
415.2
403.3
221.0
109.0
148.0
231.0
318.3
385.3
280.0
471.1
453.2
465.3
112.0
145.0
210.0
259.0
274.0
578.7
708.2

Figure S12

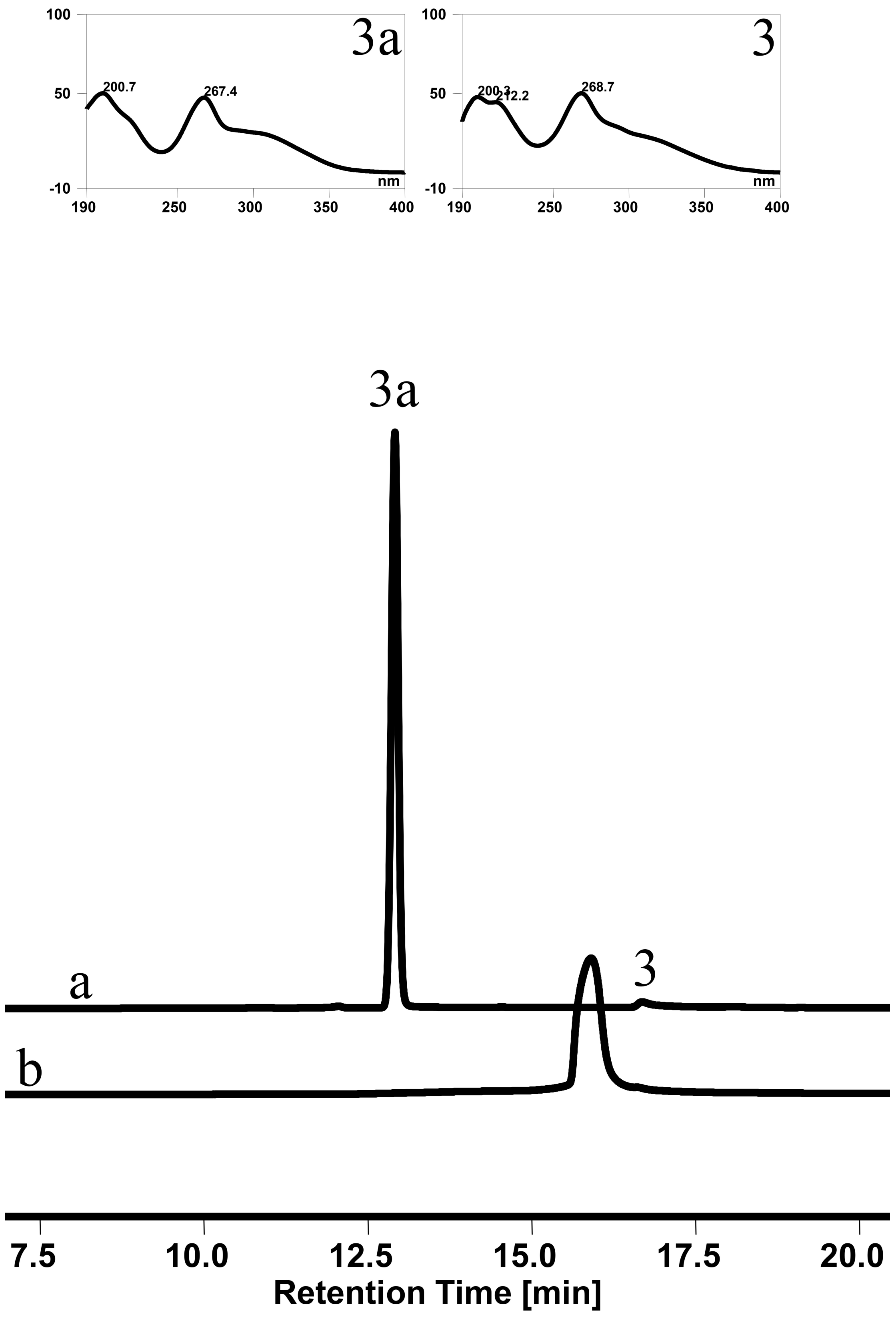
100
50
-10
200.7
267.4
3a
nm
190
250
300
350
400
100
50
-10
212.2
268.7
3
nm
190
250
300
350
400
3a
3
a
b
7.5
10.0
12.5
15.0
17.5
20.0
Retention Time [min]

Figure S13

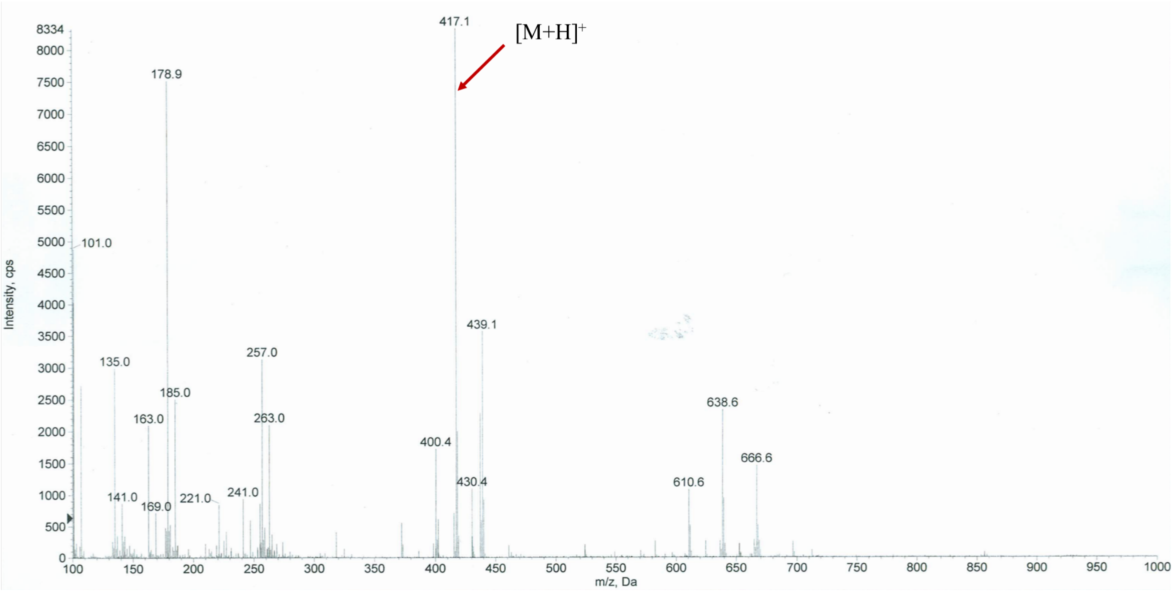

[M+H]+
417.1
178.9
101.0
439.1
135.0
257.0
185.0
163.0
263.0
400.4
141.0
169.0
221.0
241.0
430.4
610.6
638.6
666.6
Intensity, cps
m/z, Da

Figure S14

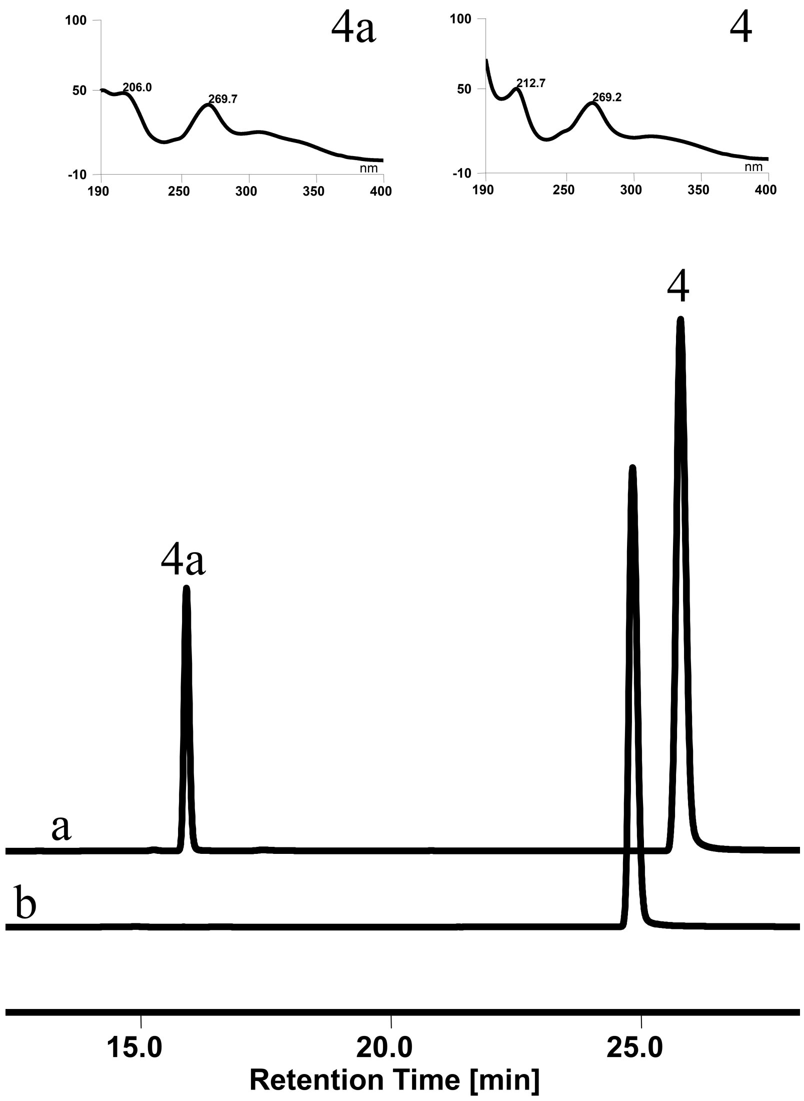

Figure S15

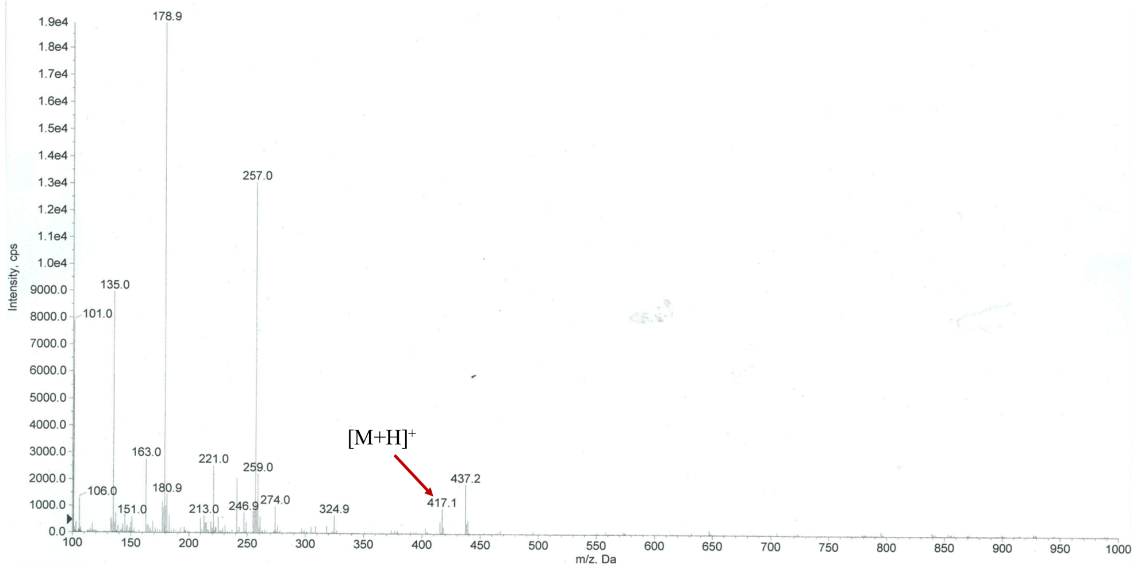

Figure S16

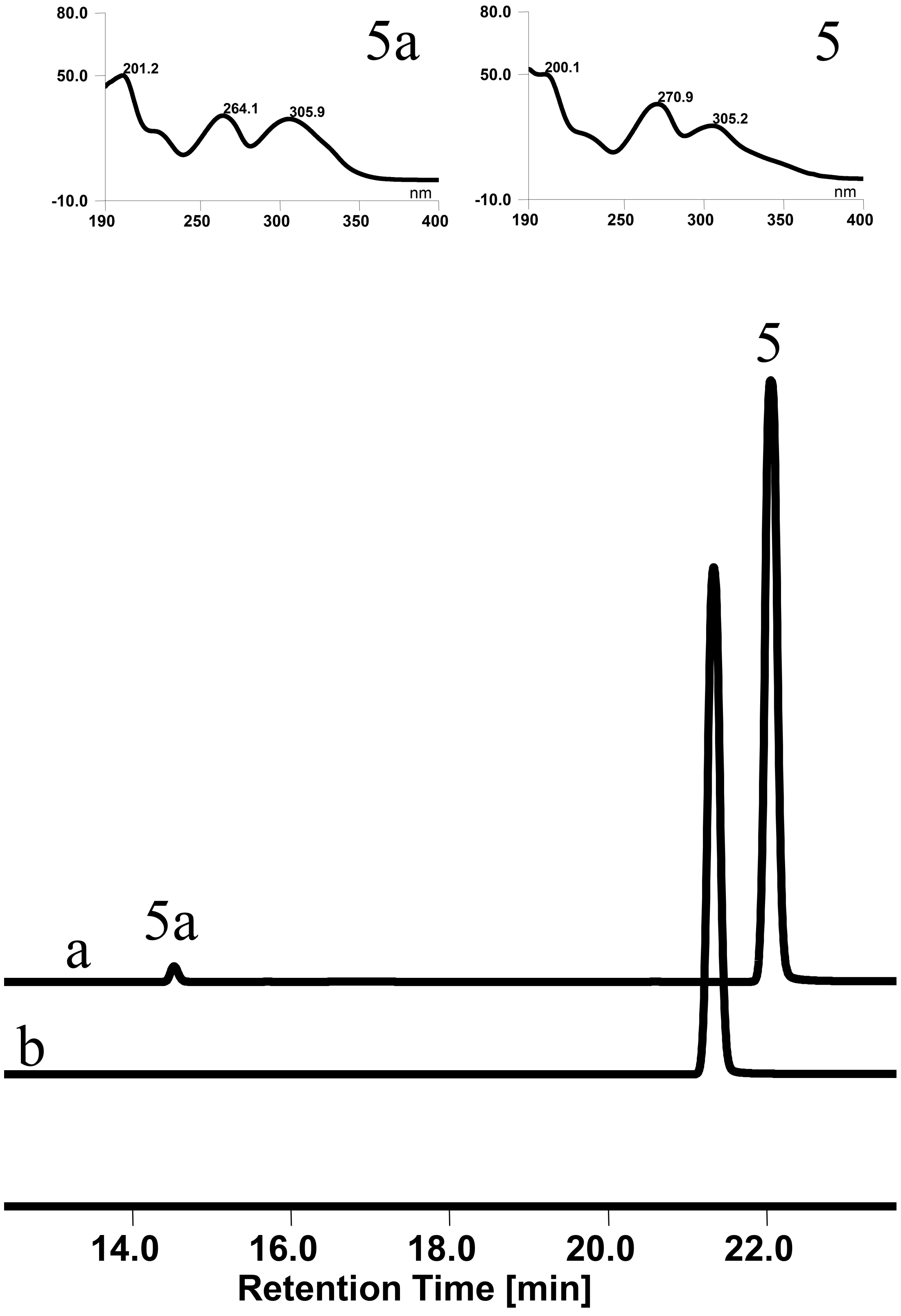
80.0
50.0
-10.0
201.2
264.1
305.9
5a
nm
190
250
300
350
400
80.0
50.0
-10.0
200.1
270.9
305.2
5
nm
190
250
300
350
400
5
5a
a
b
14.0
16.0
18.0
20.0
22.0
Retention Time [min]

Figure S17

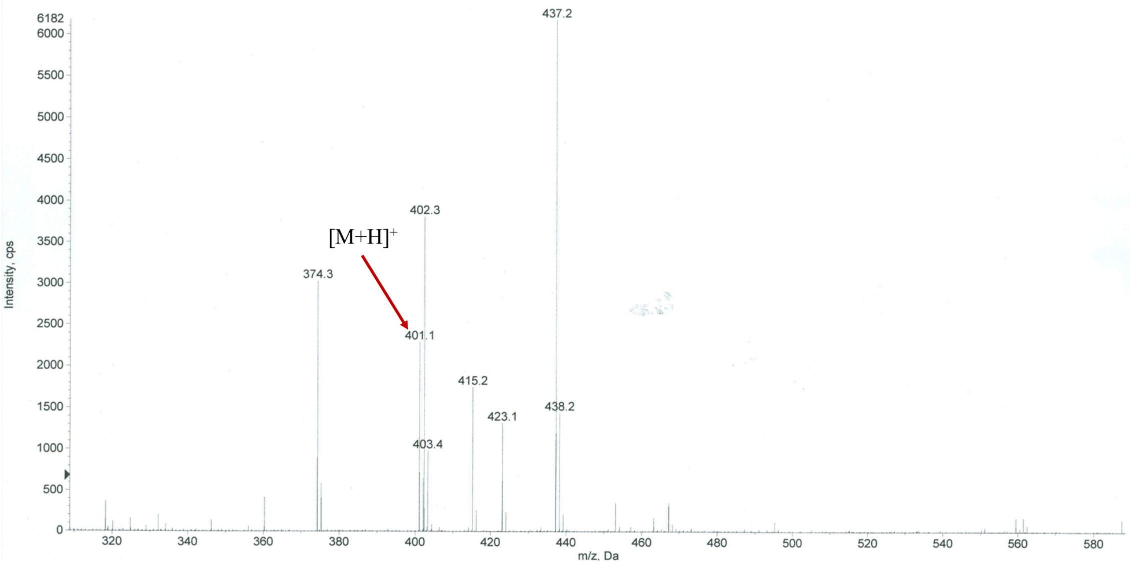

6182
6000
5500
5000
4500
4000
3500
3000
2500
2000
1500
1000
500
0
Intensity, cps
437.2
402.3
[M+H]+
374.3
401.1
415.2
423.1
438.2
403.4
320
340
360
380
400
420
440
460
480
500
520
540
560
580
m/z, Da

Figure S18

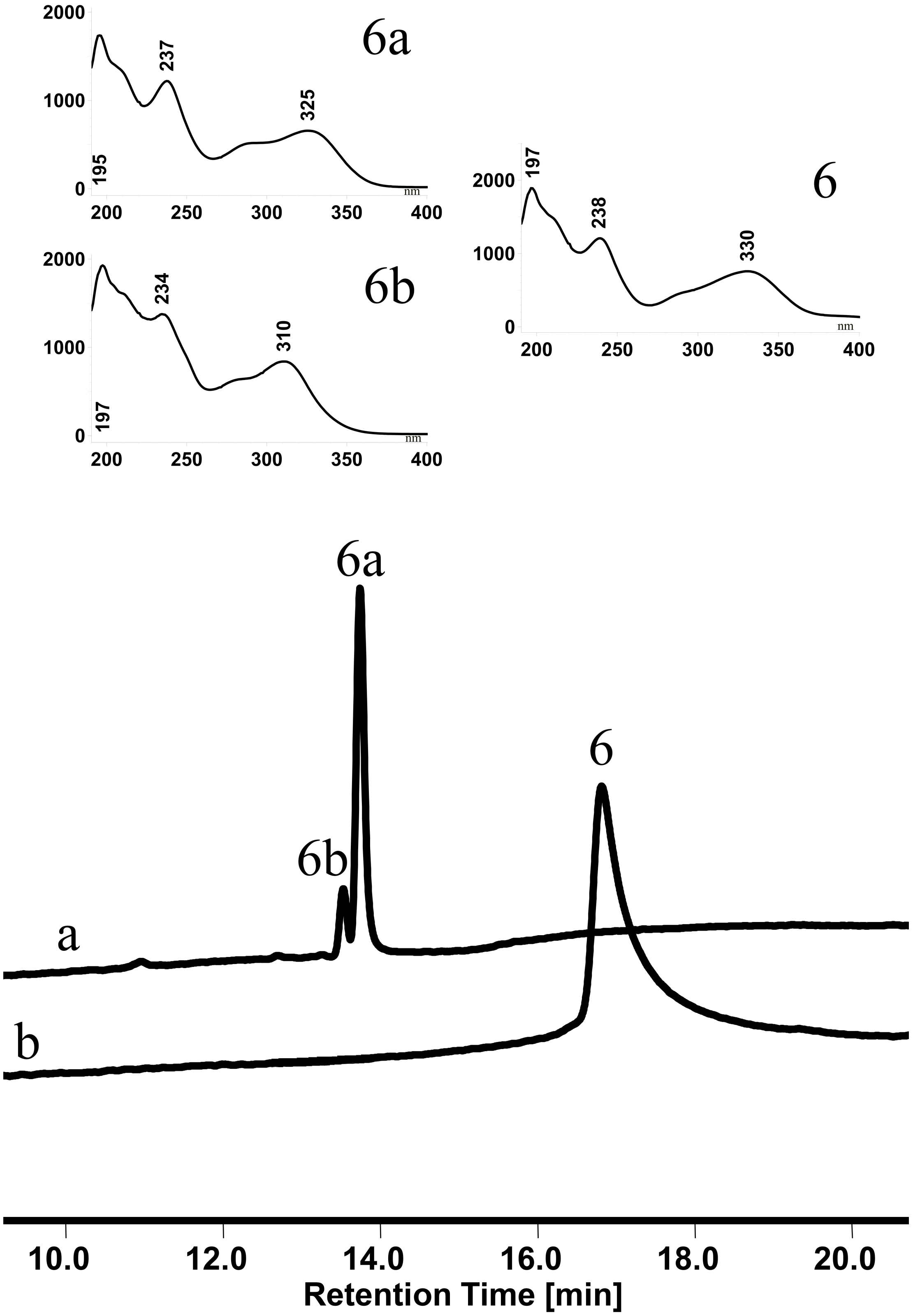
6a
2000
1000
0
237
325
195
nm
200 250 300 350 400
6b
2000
1000
0
234
310
197
nm
200 250 300 350 400
6
2000
1000
0
197
238
330
nm
200 250 300 350 400
6a
6b
6
a
b
10.0 12.0 14.0 16.0 18.0 20.0
Retention Time [min]

Figure S19

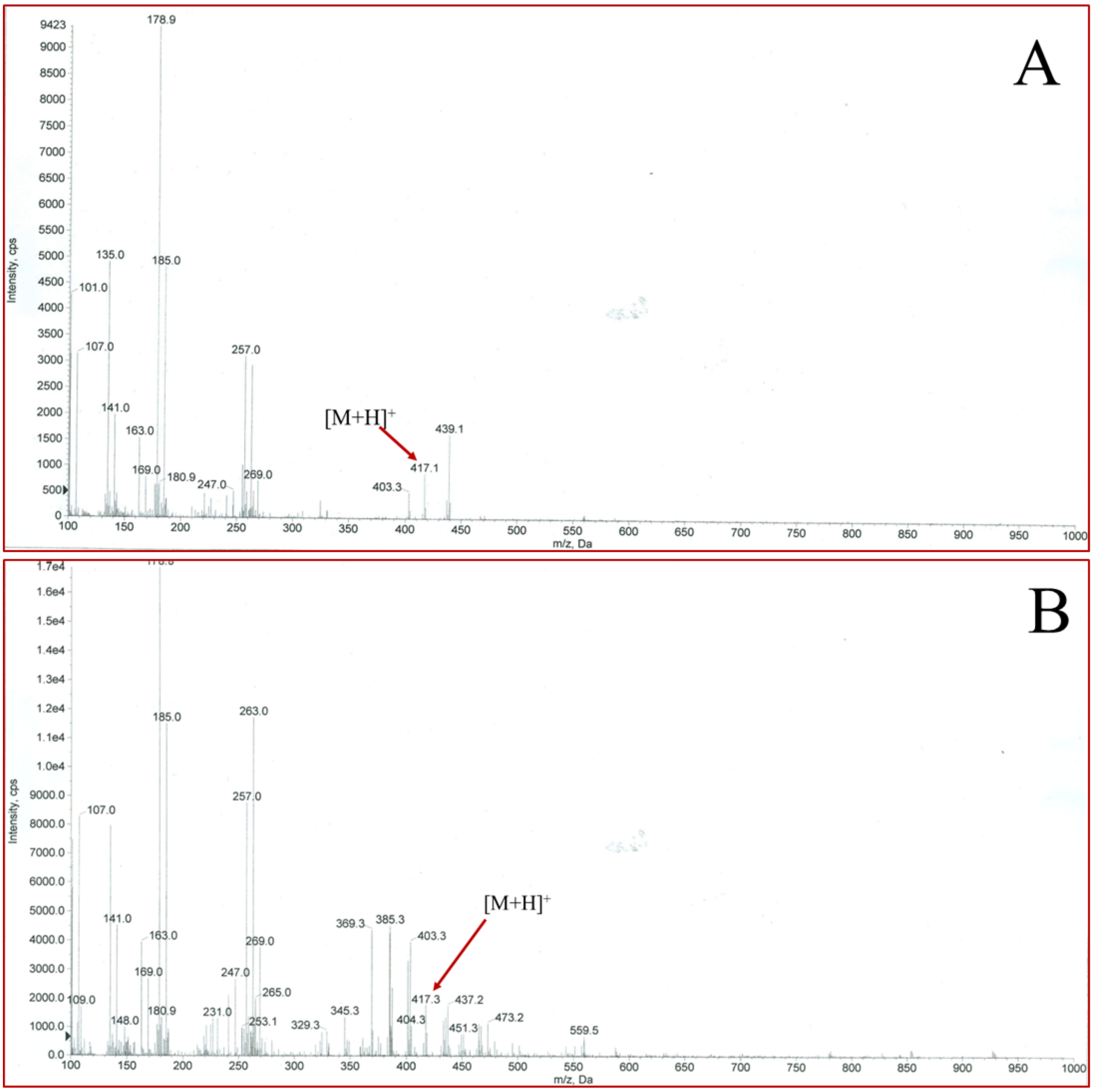

A
B
[M+H]+
[M+H]+
Intensity, cps
m/z, Da
178.9
135.0
185.0
101.0
107.0
257.0
141.0
163.0
439.1
169.0
180.9
247.0
269.0
417.1
403.3
263.0
369.3
385.3
403.3
417.3
437.2
109.0
148.0
231.0
253.1
265.0
329.3
345.3
404.3
451.3
473.2
559.5

Figure S20
